# Ropivacaine inhibits wound healing by suppressing the proliferation and migration of keratinocytes via the PI3K/AKT/mTOR Pathway

**DOI:** 10.1186/s12871-022-01646-0

**Published:** 2022-04-15

**Authors:** Xiaoyang Wu, Quanyu Sun, Simeng He, Ya Wu, Shihan Du, Lirong Gong, Jianbo Yu, Haifeng Guo

**Affiliations:** 1 School of Medicine, Nankai University, Tianjin, China; 2 the First Clinical Medical College, Mudanjiang Medical University, Mudanjiang, , Heilongjiang China; 3 Department of Anesthesiology and Critical Care Medicine, Tianjin Nankai Hospital, Tianjin Medical University, Tianjin, China; 4 Department of General Surgery, Hongqi Hospital Affiliated to Mudanjiang Medical University, Mudanjiang, Heilongjiang China

**Keywords:** Ropivacaine, Wound healing, PI3K, AKT, mTOR Pathway

## Abstract

**Background:**

After surgery, millions of people suffer from delayed healing or wound dehiscence with subsequent severe complications, even death. Previous studies have reported that ropivacaine exhibits anti-proliferative and anti-migratory activities on numerous cells. Whether ropivacaine is able to influence the proliferation and migration of keratinocytes is still unclear. This study aimed to investigate the effect of ropivacaine on keratinocytes and its underlying molecular mechanism.

**Methods:**

Adult male Sprague–Dawley rats were allocated to establish wound healing models with or without 0.75% ropivacaine treatment and assessed the epidermal thickness by HE staining. HaCaT cells were cultured to evaluate the effect of ropivacaine on wound healing. The cell proliferation, apoptosis status and migration were detected in vitro. Moreover, western blotting was used to examine expression to with PI3K/AKT/mTOR signaling pathways for molecular studies and the changes in inflammatory factors (IL-6, IL-10, TNF-α) were detected by ELISA.

**Results:**

In the present study, we found that ropivacaine delayed wound closure in vivo. In vitro experiments, it was demonstrated that ropivacaine significantly inhibited the proliferation and migration of HaCaT cells via the suppression of PI3K/AKT/mTOR signaling pathway. Activation of PI3K/AKT/mTOR signaling pathway reversed the effects of ropivacaine on the proliferation and migration of HaCaT cells. Furthermore, ropivacaine contributed to the release of pro-inflammatory cytokines (IL-6 and TNF-α) and inhibited the secretion of anti-inflammatory cytokines of keratinocytes (IL-10).

**Conclusions:**

Our research demonstrated that ropivacaine treatment showed a more decreased wound closure rate. Mechanistically, we found that ropivacaine suppressed the proliferation and migration of keratinocytes and altered the expression of cytokines by inhibiting PI3K/AKT/mTOR pathway.

**Supplementary Information:**

The online version contains supplementary material available at 10.1186/s12871-022-01646-0.

## Background

Wound healing is a highly complex multi-step process influenced by various factors, including multiple cellular and paracellular pathways [1–4]. Normal wound healing consists of 4 phases: hemostasis, inflammation, proliferative, and remodeling. During the healing process, keratinocytes are critical for re-establishing the exposed epithelium and facilitating wound closure and skin barrier function [5]. After trauma, keratinocytes are activated to participate in the re-epithelialization through cell migration and proliferation [6]. Enhanced migration of keratinocytes drives the spontaneous healing process, while delayed migration indicates a negative effect.

Local anesthetics (LAs) are a group of commonly used drugs for pain relief. Infiltration of the surgical incision site with long-acting LAs at the end of the operation is a common clinical practice for postoperative analgesia [7]. Since the direct contact between the LAs and the surgical wounds, a growing concern has been recently attracted to the potential adverse effects of long-acting LAs on wound healing. Ropivacaine is considered one of the safest long-acting LAs due to its low toxicity in the cardiovascular and central nervous systems and is the most widely used in postoperative incision infiltration [8–10]. Previous studies have reported that concentrations as low as 10 μM have a dose-dependent inhibitory effect on the proliferation of fibroblasts and tenocytes [11]. These observations are worrisome. Even low concentrations of ropivacaine can weaken the wound and delay its healing. However, the effect of ropivacaine on keratinocytes needs further exploration.

PI3K is an intracellular phosphatidylinositol kinase and AKT is a serine/threonine-specific protein kinase. PI3K/AKT/mTOR pathway plays a crucial role in the proliferation, migration, metabolism and apoptosis of various cells [12–14]. Recent experimental studies have shown that ropivacaine decreased the growth, migration and invasion of gastric cancer cells by inactivating the PI3K/AKT/mTOR signaling pathway [15]. In addition, ropivacaine inhibits the apoptosis and proliferation of chronic myeloid leukemia cells in a dose- and time-dependent manner by suppressing the PI3K/AKT/mTOR pathway [16]. However, whether ropivacaine participates in wound healing, through the PI3K/AKT/mTOR signaling pathway, remains unknown.

In this work, we investigated the effects of ropivacaine on wound healing. In vitro and in vivo studies, we found that the potential mechanism of action of ropivacaine in delayed wound healing is inhibition of the PI3K/AKT/mTOR pathway in keratinocytes.

## Methods

### Animal model for wound healing

In a total of 10 Sprague–Dawley rats (male, 200–250 g, 4-week-old) were purchased from Vital River (Beijing, China). All rats were housed at 25 °C with 12 h light and dark photocycle, food and water supplied. They were randomly divided into two groups (*n* = 5 per group), control and ropivacaine groups. All rats were first anesthetized with 2% ~ 3% isoflurane (R510-22, RWD Life Science, China) inhalation for induction and 1.5% isoflurane for maintenance. The back fur of the rats was carefully shaved for the area assigned for wounding. Excisional wounds with the size of 1 cm × 1 cm were created on each rat at day 0. A total of 1 ml containing 0.75% ropivacaine (ropivacaine group) or PBS (control group) was injected into the surrounding tissue of the wound once a day for 10 days. The wound areas were measured every 2 days. The rate of wound closure was calculated as follows: wound closure (%) = [(Day 0 wound area − Day X wound area)/Day 0 wound area] × 100%. All experimental procedures were approved by the Animal Ethics Committee of the Tianjin Nankai Hospital (Approval No. IRM-DWLL-2019042) and were performed in accordance with the National Institutes of Health “Guidelines for the Care and Use of Laboratory Animals”.

### Hematoxylin–eosin staining and histological analysis

Ten days later, rats were sacrificed and full-thickness skin samples were obtained for histological analysis. Tissue samples were immediately collected and fixed overnight with 4% paraformaldehyde, embedded in paraffin, and subsequently sectioned at a 4 μm thickness for further hematoxylin–eosin staining according to standard procedures. HE samples were observed to assess the epidermal thickness, and the epidermal thickness was measured by ImageJ software.

### Cell culture and drug action

HaCaT cells were purchased from the Cell Center of the Chinese Academy of Sciences(Shanghai, China). Cells were cultured and maintained in RPMI1640 medium (Gibco, ThermoFisher Scientific, USA) supplemented with 10% fetal bovine serum (FBS; Gibco; ThermoFisher Scientific, USA) and 1% penicillin/streptomycin (Invitrogen, CA, USA) in an incubator at 37 °C in a humidified incubator under 5% CO2. Cells in the logarithmic growth phase were trypsinized by 0.25% trypsin (Hyclone, USA) and used for the following experiments. Ropivacaine (Naropen, AstraZeneca, Sweden) was dissolved in sterile physiological saline (0.9% NaCl) and diluted to the given concentration. Cells were treated with 2.5–10 μmol/ L of ropivacaine for 6-24 h and then subjected to other experiments.

### CCK-8 assay

HaCaT cells were trypsinized and resuspended at a density of 2 × 10^3^/mL, then inoculated in a 96-well plate with 100 μL per well. Afterward, the 96-well plate was placed in an incubator for further culture. The 96-well plate was then placed into an incubator for further culture. After 6, 12, 24, and 48 h of incubation, 10 μL of CCK-8 reagent (Dojindo, Japan) was added to each well and incubated for 2 h. Subsequently, the microplate reader measured the absorbance (OD value) of each well at 450 nm.

### Flow cytometry

Cell apoptosis assay was detected by flow cytometry using an Annexin V-fluorescein isothiocyanate (FITC) / propidium iodide (PI) apoptosis kit (Absin, Shanghai, China) according to the manufacturer’s instruction. Briefly, HaCaT cells were harvested using trypsin and washed with ice-cold PBS after incubation for 48 h. After that, the harvested cells were resuspended in a 300 μl binding buffer and successively double-stained with Annexin V-FITC (5 μl, 15 min) and PI (5 μl, 5 min) in the dark at room temperature. The cells were subjected to apoptosis analysis using flow cytometry.

### Wound-healing assay

HaCaT cell migration was analyzed using a wound-healing assay. Briefly, cells were cultured in 6-well plates and grown to 100% confluence. A scratch wound was created using a 200-µl pipette tip and the cells were treated with ropivacaine based on the experimental requirements for 24 h. Images were captured under a microscope (× 100; Olympus, Tokyo, Japan) and the percentage of wound healing area was calculated using ImageJ software.

### Transwell assay

Transwell assay was performed by Corning Transwell Kit. Initially, HaCaT cells were incubated in the upper chamber with 100 μL serum-free growth medium in a 24-well plate (1 × 10^6^ cells/well). 600 μl of medium containing 30% FBS was added to each lower chamber. Thereafter, PBS or the required concentration of ropivacaine were added into the lower chamber. After 24 h of cell incubation at 37 °C, the migrated HaCaT cells were fixed by the mixture of formaldehyde and acetic acid for 15 min, then washed with PBS, stained with 0.1% crystal violet. Finally, images were obtained under a microscope (× 10; Olympus, Tokyo, Japan) and the number of migrated HaCaT cells was counted using Image-Pro Plus 6.0 software.

### Western blot

For evaluation of PI3K/AKT/mTOR pathways, the proteins were extracted from the treated cells using a total protein isolation kit (Thermo Fischer Scientific, USA) and their concentrations were determined by the BCA protein assay kit (Sigma, USA). Equal quantities (30 µg/lane) of protein were separated on 5–12% SDS-PAGE gel and then were transferred to a PVDF membrane (0.2 µM, Bio-Rad, USA). After blocking with 5% skim milk in TBST for 3 h and incubated overnight at 4 °C with primary antibody against PI3K (ab32089; 1:1,000; 110 kDa), p-PI3K (ab182651; 1:1,000; 84 kDa), AKT (ab8805; 1:500; 57 kDa), p-AKT (ab38449; 1:500; 56 kDa), mTOR (ab2732; 1:2,000; 289 kDa), p-mTOR (ab109268; 1:2,000; 289 kDa) and GAPDH (ab181602; 1:5,000; 36 kDa). Subsequently, the membranes were washed with TBST 5 times (5 min for each time) and then incubated with HRP-labeled secondary antibodies (CST7074; 1:2,000) for 1 h at room temperature. The blots were visualized using an enhanced chemiluminescence Western blot detection kit (170–5070, Bio-Rad, USA), and the relative expression of target proteins was quantified by the Image-Analysis system. Membranes were cut out before blotting, and therefore full-length blots were not available. Uncropped images were shown in Supplementary Files.

### Reverse transcription-quantitative polymerase chain reaction (RT-qPCR)

Total RNA was extracted from cultured HaCaT cells treated with or without ropivacaine using an RNeasy Mini Kit (Qiagen, Hilden, Germany) in accordance with the manufacturer’s manuals. Isolated RNA (500 ng) was converted into cDNA using Prime-Script RT reagent Kit (TaKaRa Biotechnology Co., Ltd., China) under the conditions: 37 °C for 15 min, 85 °C for 5 s, and 4 °C for 10 min using the T100 Thermal Cycler (Bio-Rad, USA). RT-PCR was performed using the 7500 real-time PCR system (Applied Biosystems, USA) with the specific primers of IL-6: forward 5′-CCACTGCCTTCCCTACTTCA-3′, reverse 5′-TCTTGGTCCTTAGCCACTCC-3′; IL-10: forward 5ʹ-CGCTGTCACCGCTTCTTCA-3ʹ, reverse ʹ-TCCCGTTCTCATCCATCTTCTC-3ʹ; TNF-α: forward 5′-TCTTCTCATTCCTGCTCGTG-3′, reverse 5′-GAGGCTGACTTTCTCCTGGT-3′; GAPDH, forward 5′-ATGGGAAGCTGGTCATCAAC-3, reverse 5′-GGATGCAGGGATGATGTTCT-3′. PCR conditions were as follows: pre-degeneration was performed at 95 °C for 30 s, 40 cycles of denaturation at 95 °C for 5 s, annealing and extension at 60 °C for 34 s. The relative mRNA levels for the specific genes were normalized to GAPDH mRNA and calculated by the 2^−ΔΔCt^ method.

#### ELISA

HaCaT cells were treated and cultured as per the above method. The culture medium was collected and centrifuged at 300 g at 4 °C for 5 min. The supernatants were gathered and the release of IL-6, IL-10 and TNF-α were measured according to the protocols of the ELISA Kits (BD Pharmingen).

### Statistical analysis

All data were presented as mean ± S.D. Differences were compared by student’s t-test between two groups or one-way analysis of variance (ANOVA) among multiple groups. Statistical analyses were carried out using GraphPad Prism 8.3.0 software (GraphPad Software, Inc., La Jolla, CA, USA) and *P* < 0.05 was accepted as statistically significant.

## Results

### Ropivacaine delayed wound closurein vivo

**Fig. 1:**
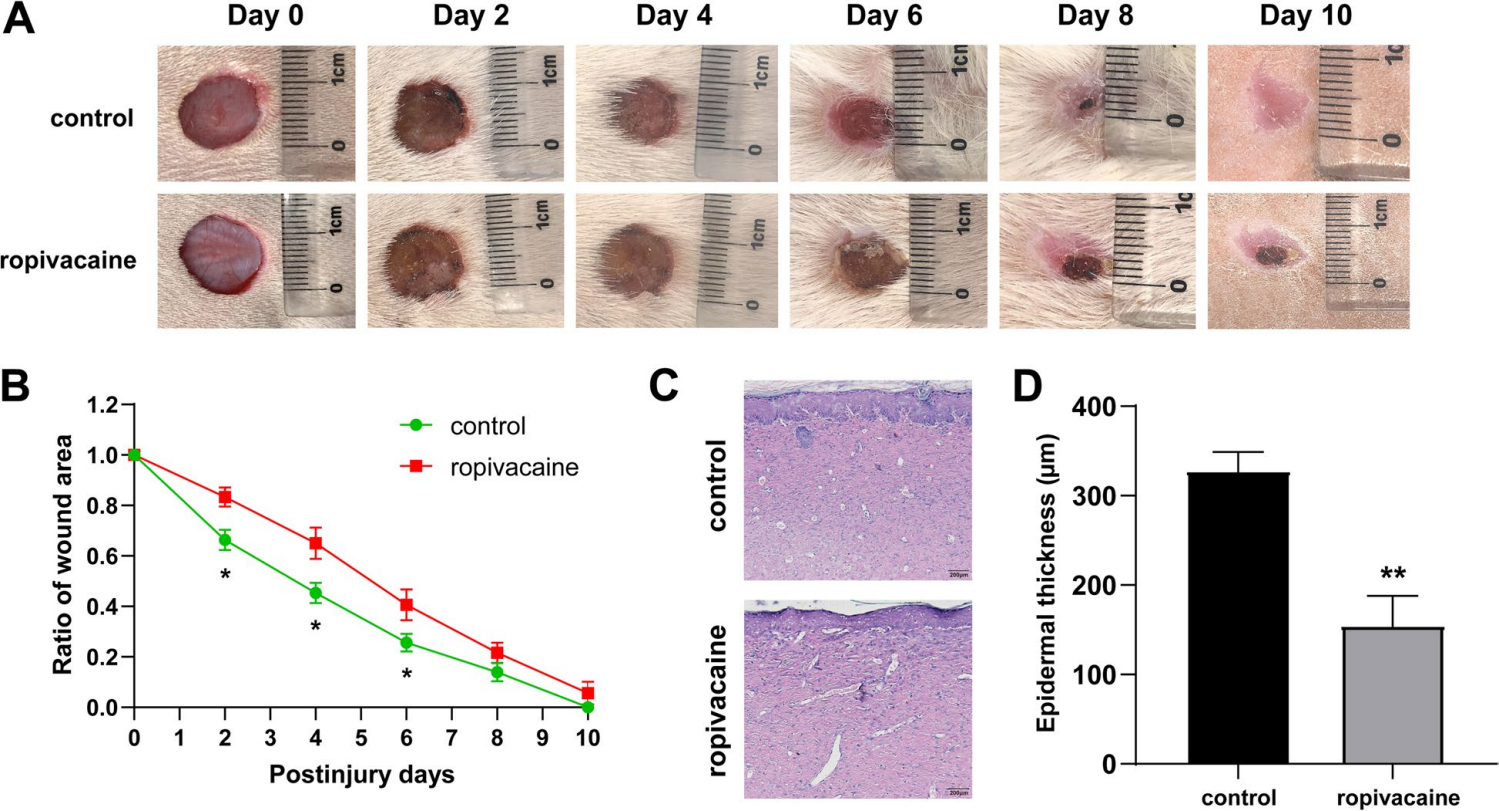
Ropivacaine treatment inhibited wound healing in mice. **A** Representative photographs showing ropivacaine treatment delayed the wound healing in the injured mouse model; **B** Quantification of the wound area gradually decreased at different time points; **C** Representative images (scale bar = 200 μm) and **D** quantification of epidermal thickness from hematoxylin and eosin-stained sections of the wound sites. Error bars represent the mean ± S.D.; *P < 0.05, **P < 0.01 vs. control

To investigate the effect of ropivacaine on wound healing in vivo, the experimental rats were divided into two groups. After creating the skin injury model in rats (as described in the Materials and method section), they were treated with PBS or ropivacaine each day for a total of 10 days. The wound area was measured every 2 days for a total of 10 days. As shown in Fig. 1A, B, the ropivacaine group showed a more decreased wound closure rate than the control group. Furthermore, we performed histopathological analysis and microscopic assessment of epidermal thickness on the final wound closure site. Compared with the control group, the epidermis of the ropivacaine group was significantly thinner (Fig. 1C, D). These findings indicate that ropivacaine impairs wound repair by inhibiting re-epithelialization.

### Ropivacaine inhibited the proliferation and induced apoptosis of HaCaT cells

**Fig. 2:**
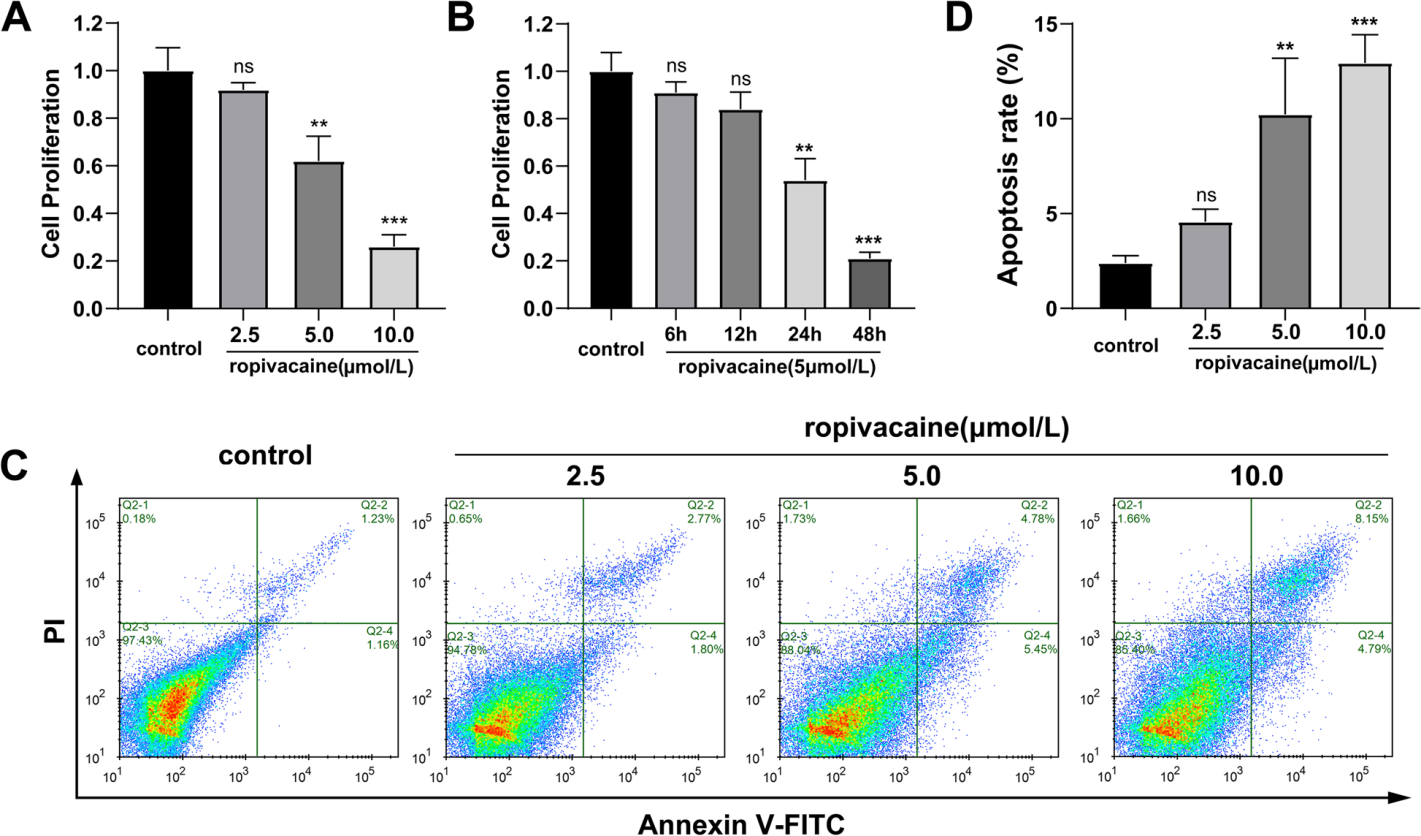
Ropivacaine inhibited the proliferation of HaCaT cells. **A** Proliferation of HaCaT cells treated with different concentrations of ropivacaine (0, 2.5, 5, 10 μmol/L) was detected by CCK8 assay; **B** Cell proliferation by CCK8 assay after treating with ropivacaine (5 μmol/L) at various time points (6, 12, 24, 48 h); **C** and **D** Subjected to ropivacaine (0, 2.5, 5, 10 μmol/L), HaCaT cell apoptosis was detected by flow cytometry. Error bars represent the mean ± S.D.; **P < 0.01, ***P < 0.001vs. control

In order to investigate the effect of ropivacaine on HaCaT cells, we initially treated it with a concentration gradient of ropivacaine (0, 2.5, 5,10 μmol/L). A CCK-8 assay demonstrated that ropivacaine significantly inhibited the proliferation of HaCaT cells in a dose- and time- dependent manner (Fig. 2A, B). Furthermore, the levels of apoptosis of HaCaT cells were investigated and we found that ropivacaine treatment significantly increased the apoptosis rate of HaCaT cells (Fig. 2C, D). Therefore, these results indicated that ropivacaine had an inhibitory effect on the proliferation of HaCaT cells in vitro, which was possibly associated with increased apoptosis.

### Ropivacaine suppressed the migration of HaCaT cells

**Fig. 3:**
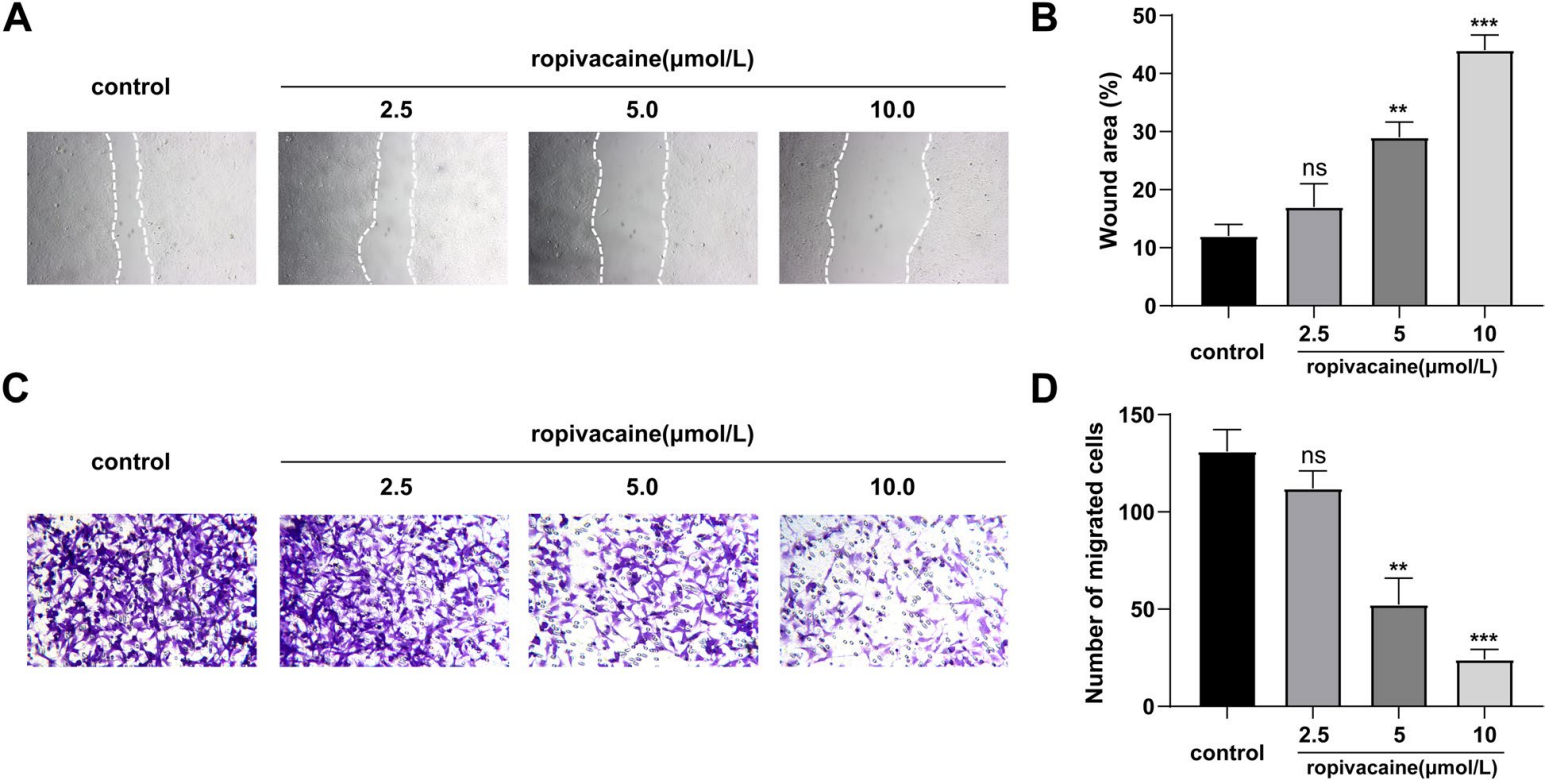
Ropivacaine inhibited the migration of HaCaT cells. **A** and **C** Typical images of scratch wound healing assays and transwell migration assays performed in HaCaT cells treated with different concentrations of ropivacaine (0, 2.5, 5, 10 μmol/L); **B** and **D** The statistical results of scratch wound healing assays and Transwell migration assays. Error bars represent the mean ± S.D.; **P < 0.01, ***P < 0.001vs. control

Next, we investigated the effects of ropivacaine on the migration ability of HaCaT cells via both a scratch wound healing assay and a transwell assay. Results of these assays indicated that ropivacaine significantly inhibited HaCaT cells migration compared with the control group (Fig. 3A-D). Furthermore, we found that with increasing ropivacaine concentration, there is increasing suppression of HaCaT cells migration (Fig. 3A-D). These data indicate that ropivacaine decreased HaCaT cells migration in a dose-dependent manner.

### Ropivacaine inhibited the activation of PI3K/AKT/mTOR pathway in HaCaT cells

**Fig. 4:**
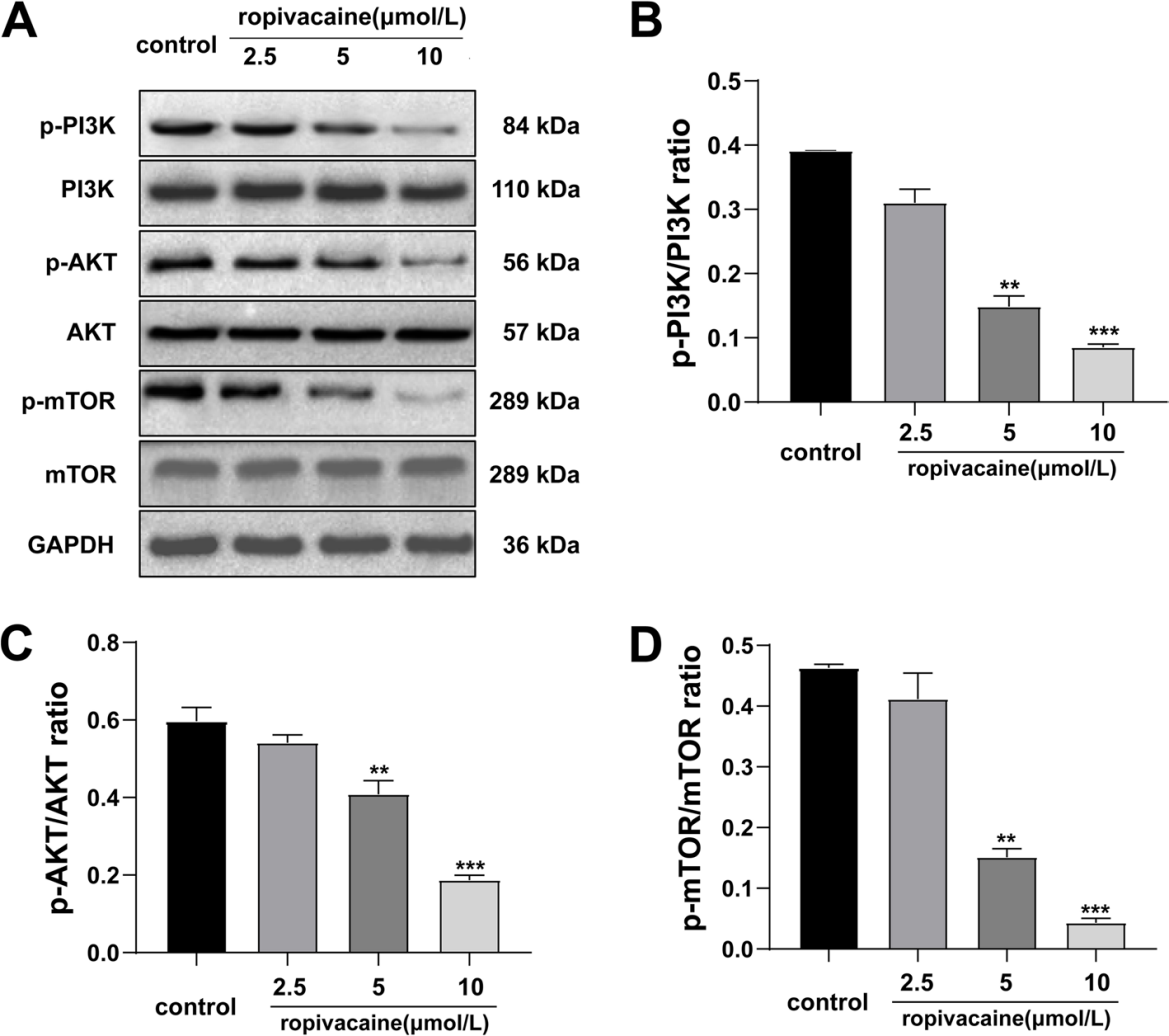
Effects of different concentrations of ropivacaine on the expression levels of important proteins for PI3K/AKT/mTOR signaling pathway in HaCaT cells. **A** Western blotting of the indicated proteins in HaCaT cells treated with 0, 2.5, 5, 10 μmol/L for 24 h; **B**-**D** Relative quantification analysis of p-PI3K/PI3K, p-AKT/AKT, and p-mTOR/mTOR. Blots were cut around specified molecular weights prior to separate probing with specific antibodies. Unedited blots are shown in the Supplementary File. Error bars represent the mean ± S.D.; **P < 0.01, ***P < 0.001vs. control

There have been many studies on the inhibition of PI3K/AKT signaling pathway of ropivacaine in vivo and in vitro [15–17]. Meanwhile, activation of the PI3K/AKT/mTOR signaling pathway has been confirmed to be closely related to the proliferation and migration of human keratinocytes [18–20]. Thus, we speculated that ropivacaine may suppress keratinocyte proliferation and migration via the PI3K/AKT signaling pathway, affecting healing adversely. As expected, western blot analysis showed that the activities of PI3K, AKT, and mTOR were modulated by ropivacaine (Fig. 4A). Compared to controls, following treatment with ropivacaine, the levels of phospho-PI3K, phospho-AKT and phospho-mTOR were significantly decreased in a dose-dependent manner (Fig. 4B-D).

### The PI3K/AKT/mTOR signaling pathway was involved in the ropivacaine-mediated suppression of HaCaT cells proliferation and migration

**Fig. 5:**
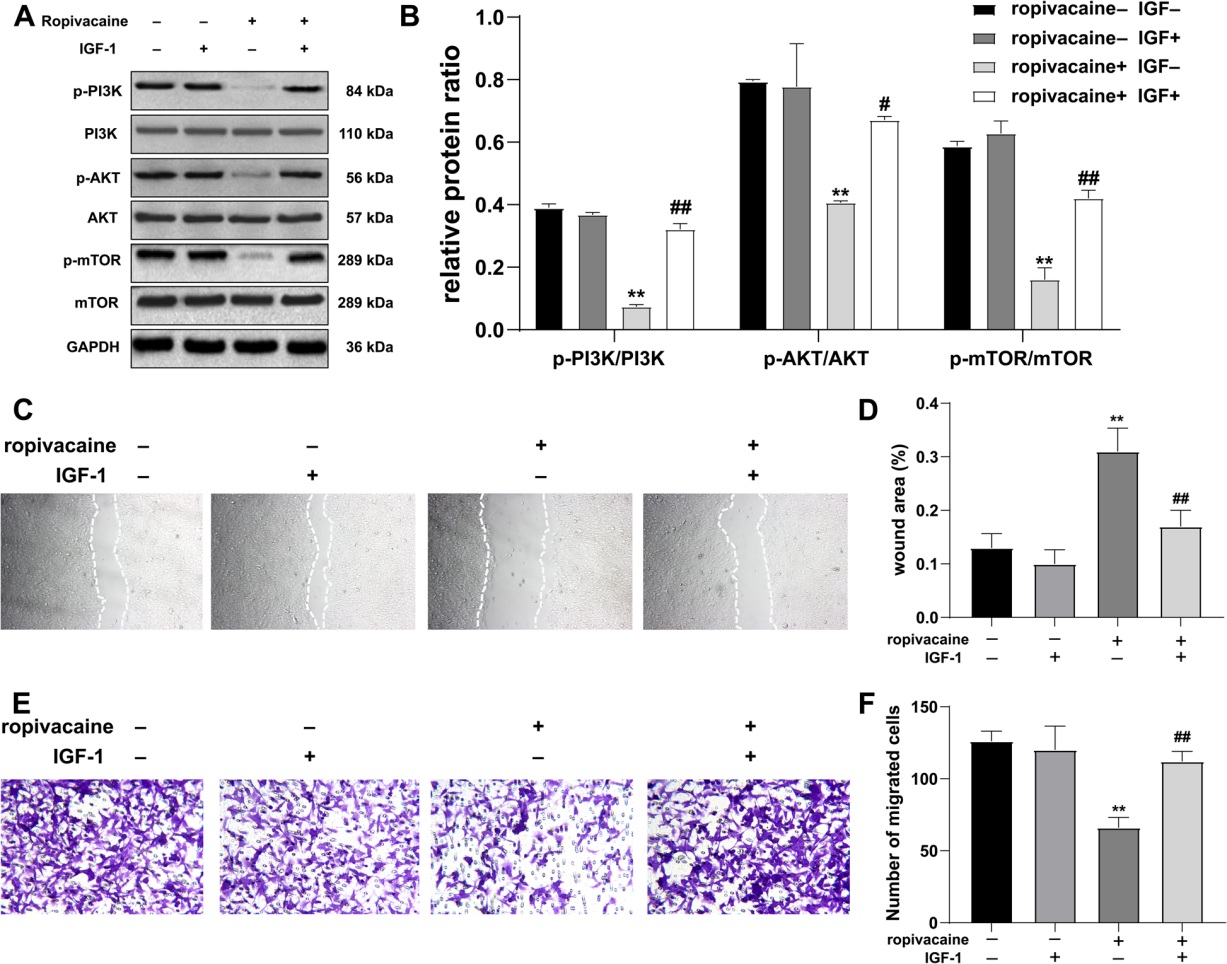
Ropivacaine inhibits the proliferation and migration of HaCaT cells by suppressing the PI3K/AKT/mTOR pathway. **A** Effect of ropivacaine on proteins expression related to PI3K/AKT/mTOR signaling pathways after IGF-1 intervention as determined by western blot analysis; blots were cut around specified molecular weights prior to separate probing with specific antibodies. Unedited blots are shown in Supplementary File. **B** Quantification of the western blot analysis in **A**; Typical images of wound scratch **C** and transwell assays **E** of HaCaT cells in response to ropivacaine after IGF-1 intervention; The statistical results of wound scratch **D** and transwell assays **F**. Error bars represent the mean ± S.D.; **P < 0.01 vs. ropivacaine- IGF- group, #*P* < 0.05, ##*P* < 0.01 vs. ropivacaine + IGF- group

To further confirm the involvement of the PI3K/AKT/mTOR pathway in growth and signaling, we used PI3K/AKT agonist (IGF-1) to further explore the effects of ropivacaine (5 μmol/L) on HaCaT cells. After adding IGF-1 (5 μmol/L) for 24 h, the expression level of proteins in the PI3K/AKT/mTOR signaling pathway was assessed by western blot analysis. The results suggest that IGF-1 treatment weakened the inhibitory effect of ropivacaine on the PI3K/AKT/mTOR signal pathway (Fig. 5A-B). In addition, activation of PI3K/AKT/mTOR signaling intercepted the negative effects of ropivacaine on HaCaT cells regarding migration (Fig. 5C-F). We concluded that ropivacaine inhibits keratinocytes migration via the PI3K/AKT/mTOR pathway.

### Effects of ropivacaine on inflammatory cytokine secretion in HaCaT cells

**Fig. 6:**
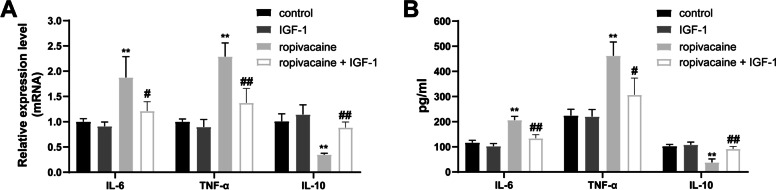
Effects of ropivacaine on the gene expression and secretion of inflammatory cytokines in HaCaT cells. **A** Relative mRNA expression levels of IL-6, IL-10 and TNF-α were evaluated by qRT-PCR in HaCaT cells; **B** The concentrations of inflammation-related cytokines in the culture supernatants was measured by ELISA. Error bars represent the mean ± S.D.; **P < 0.01 vs. control, #*P* < 0.05, ##*P* < 0.01 vs. ropivacaine group

To examine the effect of ropivacaine treatment on secreted cytokines in HaCaT cells, the mRNA expressions and concentrations of pro-inflammatory cytokines (IL-6 and TNF-α) and anti-inflammatory (IL-10) were measured in the culture supernatants. Compared with the control group, ropivacaine stimulated IL-6 and TNF-α mRNA expression and secretion and inhibited the secretion and mRNA expression of IL-10 (Fig. 6A-B). However, IGF-1 intervention remarkably reversed the alterations induced by ropivacaine (Fig. 6A-B).

## Discussion

Wound healing is subject to sophisticated regulation by a number of key factors. Failure in achieving wound closure may cause delayed healing or wound dehiscence with subsequent severe complications, even death [21, 22]. During cutaneous wound healing, keratinocytes make up approximately 90%-95% of the epidermis and act as an innate immune cell for skin homeostasis, which is considered the most important in maintaining structural integrity and chemical barrier function of the skin [5]. Also, it is well established that local anesthetic infiltration and infusion to the wound site, acting directly on the application site, is considered a valuable technique to relieve pain. Ropivacaine, a long-acting regional anesthetic, is commonly selected for postoperative analgesia. In this study, we found that ropivacaine delayed wound closure in vivo. We wanted to further explore the function of ropivacaine on the proliferation, apoptosis and migration of HaCaT cells during wound healing. Our experiments showed that ropivacaine inhibited the PI3K/AKT/mTOR signaling pathways, thus suppressing the proliferation and migration of HaCaT cells. Understanding the long-term impact of ropivacaine on wound healing may help design new therapies aiming at minimizing the risk of adverse events.

Accumulating studies have proved that ropivacaine exhibits anti-proliferative and anti-migratory activities on numerous cells [23–26]. For example, ropivacaine inhibits proliferation and promotes apoptosis of SH-SY5Y cells (human neuroblastoma cell line) in a dose- and time-dependent manner to induce neuronal injury [27]. Besides, ropivacaine also suppresses MSC proliferation and migration by reducing the expression of ICAM-1 via the IκB–NF-κB signaling pathway [28]. In addition, ropivacaine plays an anti-proliferative role in breast cancer cells through regulating the miR-27b-3p/YAP axis [29]. In our study, we found that ropivacaine promoted the apoptosis of HaCaT cells. Cell apoptosis inhibits healing. It can be seen from Figs. 2A and 2C that although ropivacaine is in 5 μmol/L showed a statistically significant effect on apoptosis. However, at the same 5 μmol/L, ropivacaine inhibited proliferation by up to 40% but only induced apoptosis by about 10%. At 10 μmol/L, ropivacaine inhibited proliferation by up to 75% but only induced apoptosis by about 13%. Therefore, the effect of ropivacaine on HaCaT cells mainly lies in proliferation rather than apoptosis. Apart from this, the study stipulated that proliferation and migration of HaCaT cells were suppressed by ropivacaine in a dose- and time-dependent manner, which may subsequently lead to delayed re-epithelialization, impeding the wound healing progresses.

Furthermore, the mechanisms of the ropivacaine-mediated suppression of proliferation and migration in HaCaT cells were further investigated. Wound healing is regulated by a complex network of signaling pathways stimulated by various alert signaling peptides [30]. The AKT family is at the center of an interconnected network of protein kinases involved in epidermal barrier formation [31]. In healthy skin, the PI3K/AKT/mTOR pathway controls several cellular processes and maintains epidermal homeostasis [32]. Dysregulation of PI3K/AKT/mTOR pathway in the skin contributes to several pathological conditions. PI3K/AKT/mTOR over-expression may contribute to uncontrolled proliferation in skin disorders, including skin cancers, psoriasis, and atopic dermatitis [32–34]. Whereas the deficient activity of PI3K/AKT/mTOR leads to severe epidermal defects, including disruption of keratinocytes integrity and delayed wound healing [35, 36]. The PI3K/AKT/mTOR signaling pathway is one of the numerous molecular signaling pathways that can affect the initiation of wound healing [37]. Notably, accumulating studies have shown that PI3K/AKT/mTOR pathway is involved in the proliferation and migration of keratinocytes [18, 38–40], indicating that it may be associated with the migration and proliferation of keratinocytes induced by ropivacaine. Our research demonstrated that ropivacaine significantly inhibited the activation of the PI3K, AKT, and mTOR proteins, as evidenced by the decreased phosphorylation of these proteins. Additionally, we found that the suppression of proliferation and migration induced by ropivacaine was significantly reversed after intervention with IGF-1, a PI3K/AKT agonist. Therefore, we further confirmed that ropivacaine promotes the proliferation and migration of HaCaT cells by inhibiting the PI3K/AKT/mTOR pathway.

The direct target of ropivacaine on the PI3K/Akt/mTOR signal pathway is multifaceted in other studies. Several studies demonstrate the PI3K/Akt/mTOR signaling pathway involved in growth and survival as the targets of ropivacaine in cells. Gong et al. [41] have shown that ropivacaine inhibits mitochondrial respiratory complex I and II activities, thereby affecting the Akt/mTOR pathway activity and inducing oxidative stress in breast cancer cells. Similarly, ropivacaine can also affect membrane proteins to affect Akt/mTOR signaling pathway. Wang et al. [26] found that ropivacaine could interact with ITGB1 protein and inhibit the expression of ITGB1 protein in colon cancer cells, thereby affecting its downstream Akt and ERK signaling pathways. Also, at the molecular level, Zhang et al. [42] have demonstrated that in the tumor xenograft experiment, ropivacaine was confirmed to inhibit tumor growth, accompanied by inhibition of the IGF-1 R/PI3K/AKT/mTOR signaling axis. Even at the transcriptional level, Zhang et al. [15] confirmed that ropivacaine inhibited the proliferation, migration, invasion and increased apoptosis of GC cells by upregulating the expression of miR-520a-3p and further regulating the WEE1 and PI3K/AKT signaling pathways. Our future experiments, such as affinity chromatography, should be used to further understand the direct molecular target of ropivacaine in HaCaT cells.

As mediators of migration and proliferation, cytokines and chemokines are involved in all wound-healing phases [43]. At the early stage, activated keratinocytes are the principal source of cytokines (e.g., IL-6, IL-10, TNF-α) acting on leukocytes, keratinocytes, and fibroblasts [44, 45]. It is generally known that IL-10 is an anti-inflammatory cytokine, while IL-6 and TNF-α are pro-inflammatory cytokines. IL-10 appears to influence the wound-healing environment by decreasing the expression of pro-inflammatory/profibrotic mediators, promoting angiogenesis [46, 47]. During wound healing, IL-6 affects fibrogenesis, angiogenesis, re-epithelialization and granulation tissue formation [48, 49]. Physiological levels of TNF-α promote cellular migration, proliferation, while persistent and elevated TNF-α is detrimental to angiogenesis and collagen fiber arrangement [46, 50]. Inflammatory cytokines are a necessary component of wound healing; however, if they persist, they may hinder the wound healing process. In the present study, we detected mRNA expression and concentrations of IL-6, TNF-α, and IL-10 in the culture supernatants and the results showed that the IL-10 mRNA level was significantly decreased in the ropivacaine-treated group, whereas the mRNA expression of IL-6 and TNF-α was significantly reduced. Following this result, the concentration of IL-6, IL-10, and TNF-α secreted from the HaCaT cells showed the same trends. These findings indicated that ropivacaine contributed to the release of pro-inflammatory cytokines and inhibited the secretion of anti-inflammatory cytokines of HaCaT cells. PI3K/AKT/mTOR pathway activation has been reported to mediate cytokine production and release and has been shown to play an essential role in wound repair [47, 51]. Hence, we performed we applied IGF-1 for estimating intervention effects. The result demonstrated that IGF-1 intervention remarkably reversed the downregulation of IL-10 and the upregulation of IL-6 and TNF-α induced by ropivacaine action, suggesting that PI3K/AKT/mTOR pathway is involved in the process of ropivacaine-induced cytokines expression alterations.

## Limitation

Further studies are still required to confirm the effect of ropivacaine on other cells tightly associated with wound healing, such as fibroblasts and endothelial cells. Also, the detailed molecular mechanisms should be further determined to better understand the direct target of ropivacaine on the PI3K/Akt/mTOR pathway.

## Conclusion

Taken together, our research demonstrated that ropivacaine treatment showed a more decreased wound closure rate. Mechanistically, we found that ropivacaine suppressed the proliferation and migration of keratinocytes and altered the expression of cytokines by inhibiting PI3K/AKT/mTOR pathway. Our research may help optimize the efficacy of ropivacaine while minimizing the risk of adverse events in patients at an increased risk for delayed wound healing.

## Supplementary Information

**Additional file 1.** 

## Data Availability

WB raw data are deposited in the Supplementary Files. The datasets generated and/or analyzed during the current study are not publicly available due to data protection but are available from the corresponding author on reasonable request.

## Abbreviations

LAs: Local anesthetics
PI3K: Phosphatidylinositol 3-kinases
p-PI3K: Phosphorylated PI3K
AKT: Protein kinase B
p-AKT: Phosphorylated AKT
mTOR: Mammalian target of rapamycin
p-mTOR: Phosphorylated mTOR
