# Supplementary material for: Ropivacaine inhibits wound healing by suppressing the proliferation and migration of keratinocytes via the PI3K/AKT/mTOR Pathway
Source: BMC Anesthesiol. 2022 Apr 15;22:106. doi: 10.1186/s12871-022-01646-0 (PMC9011930; doi:10.1186/s12871-022-01646-0)

Figure 4A p-PI3K (1)

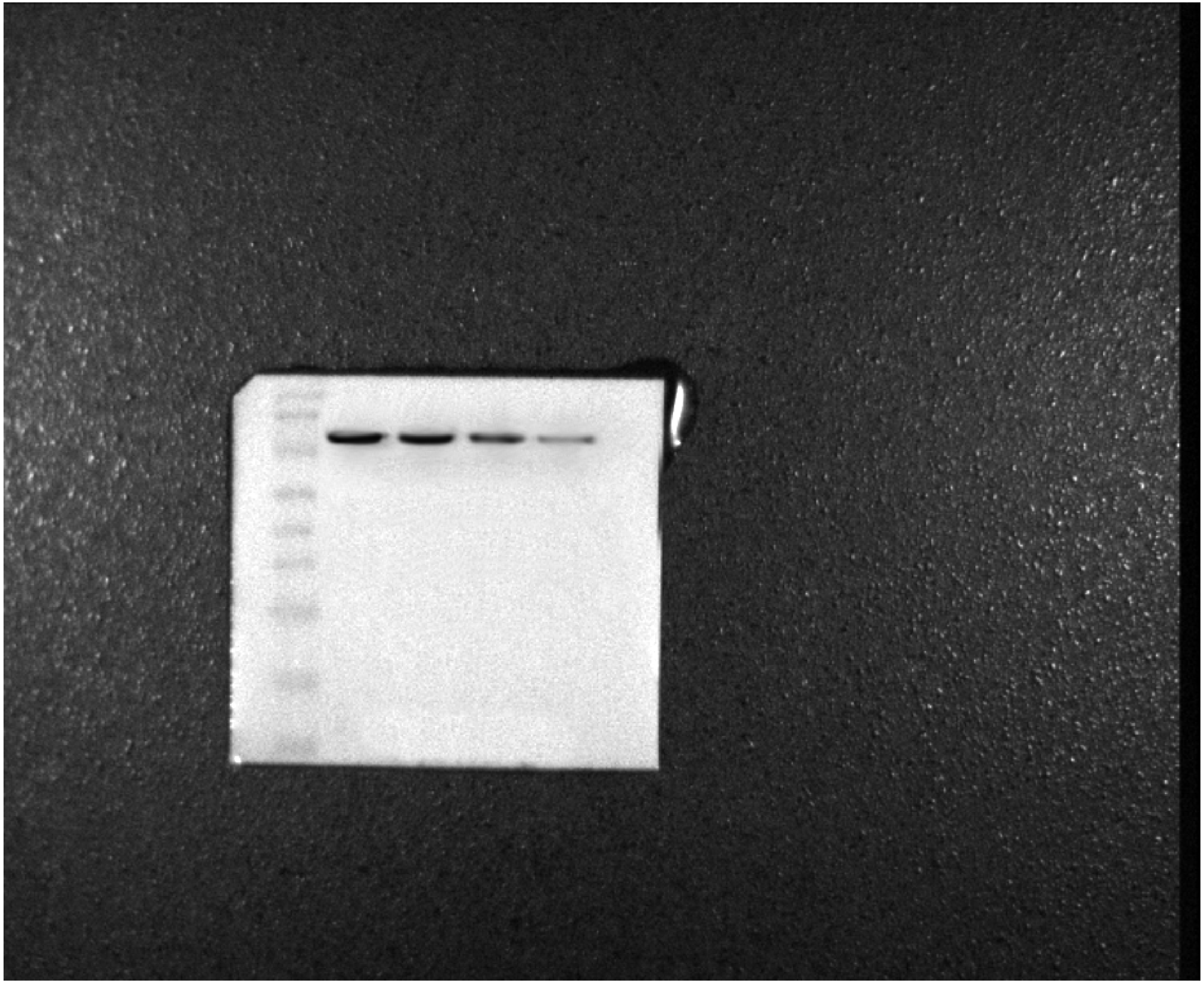

Figure 4A p-PI3K (2)

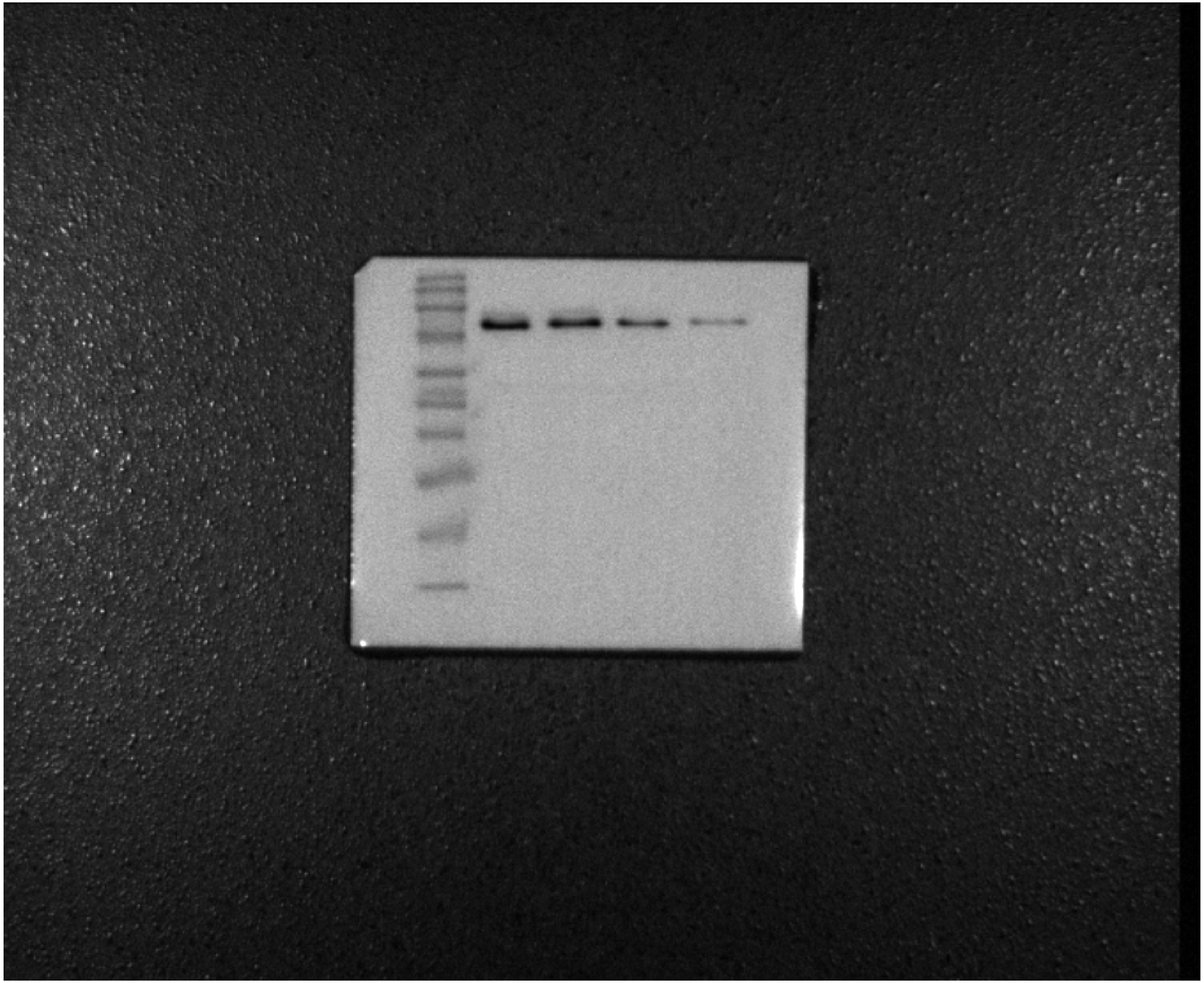

Figure 4A p-PI3K (3)

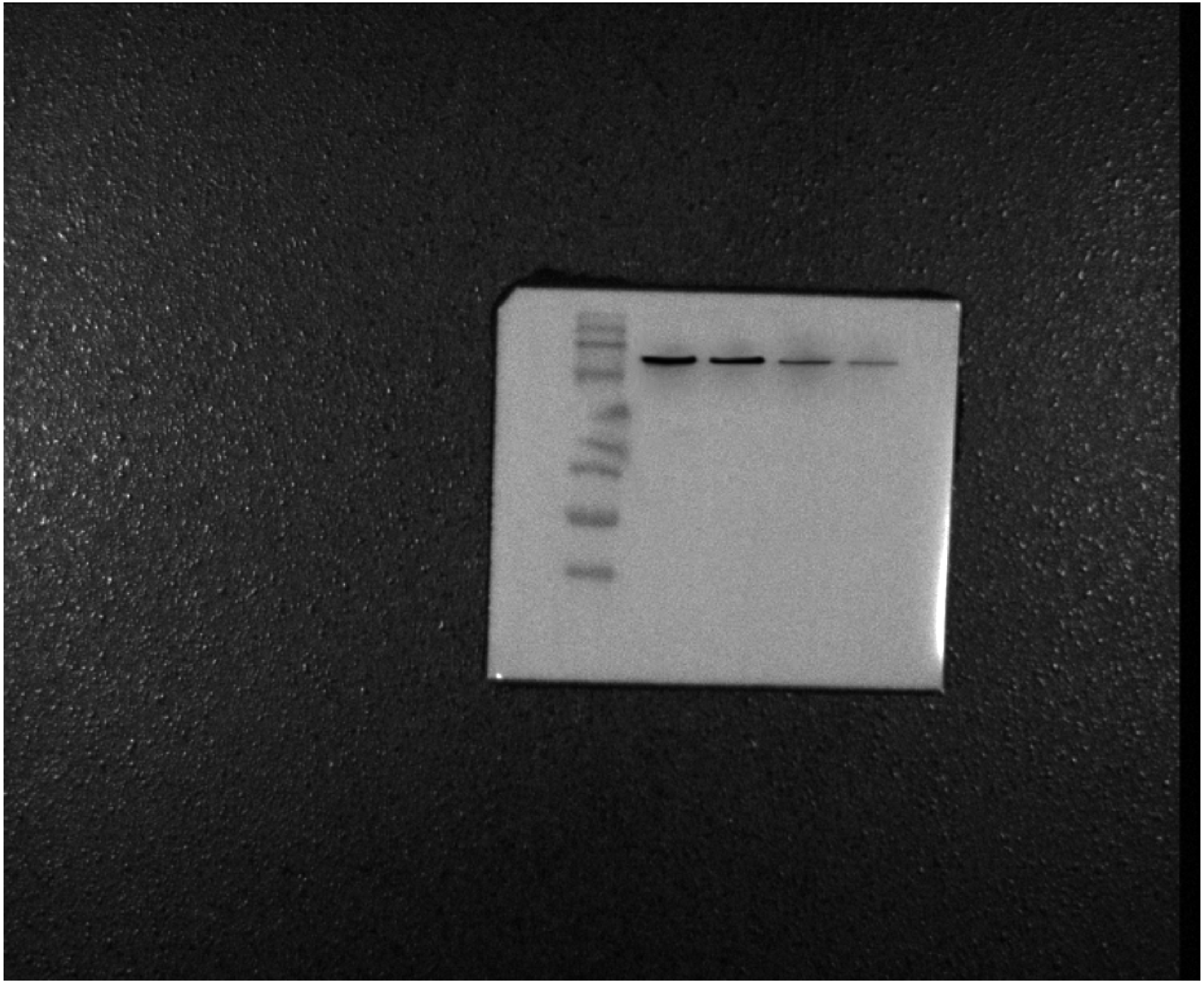

Figure 4A PI3K (1)

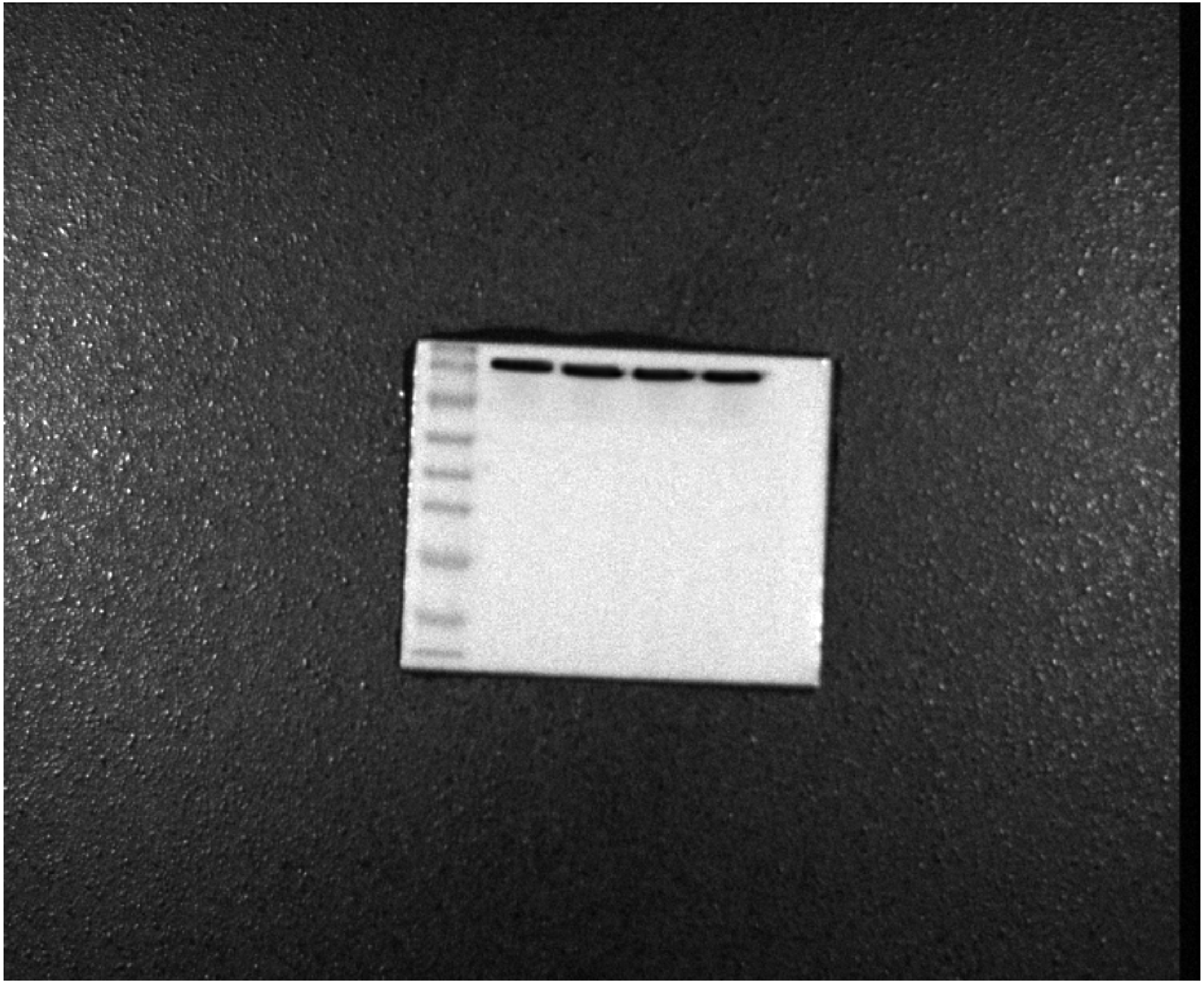

Figure 4A PI3K (2)

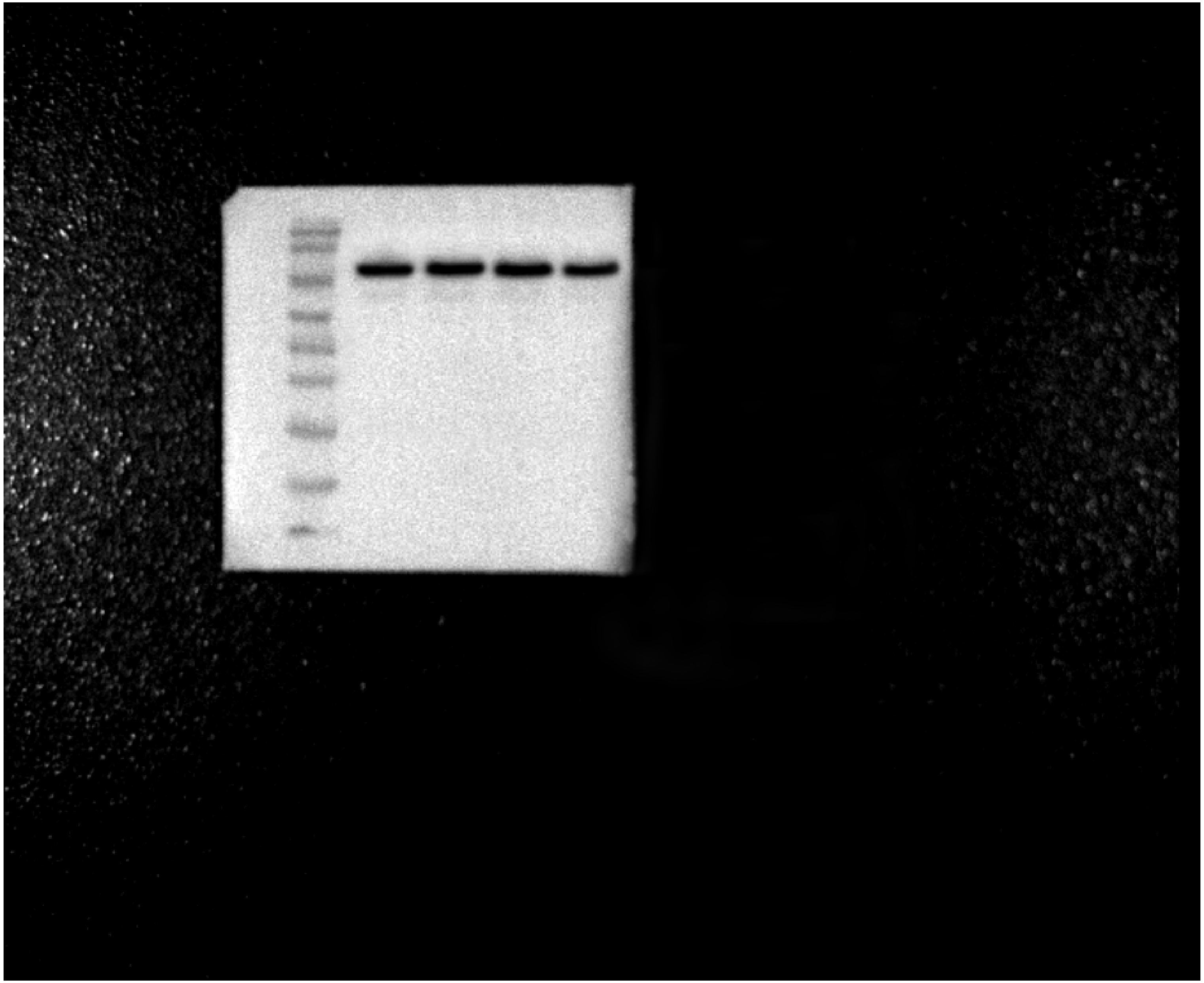

Figure 4A PI3K (3)

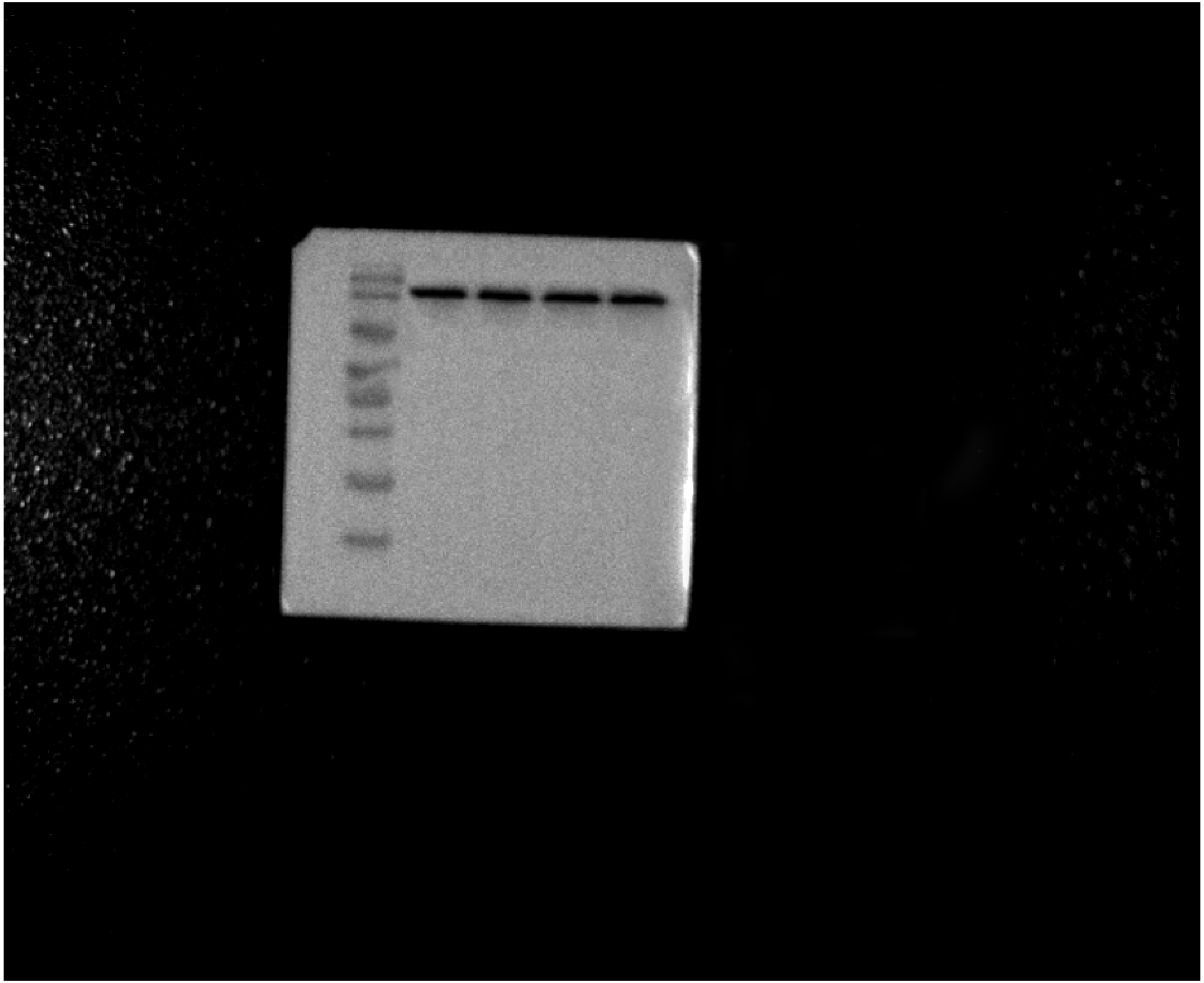

Figure 4A p-AKT (1)

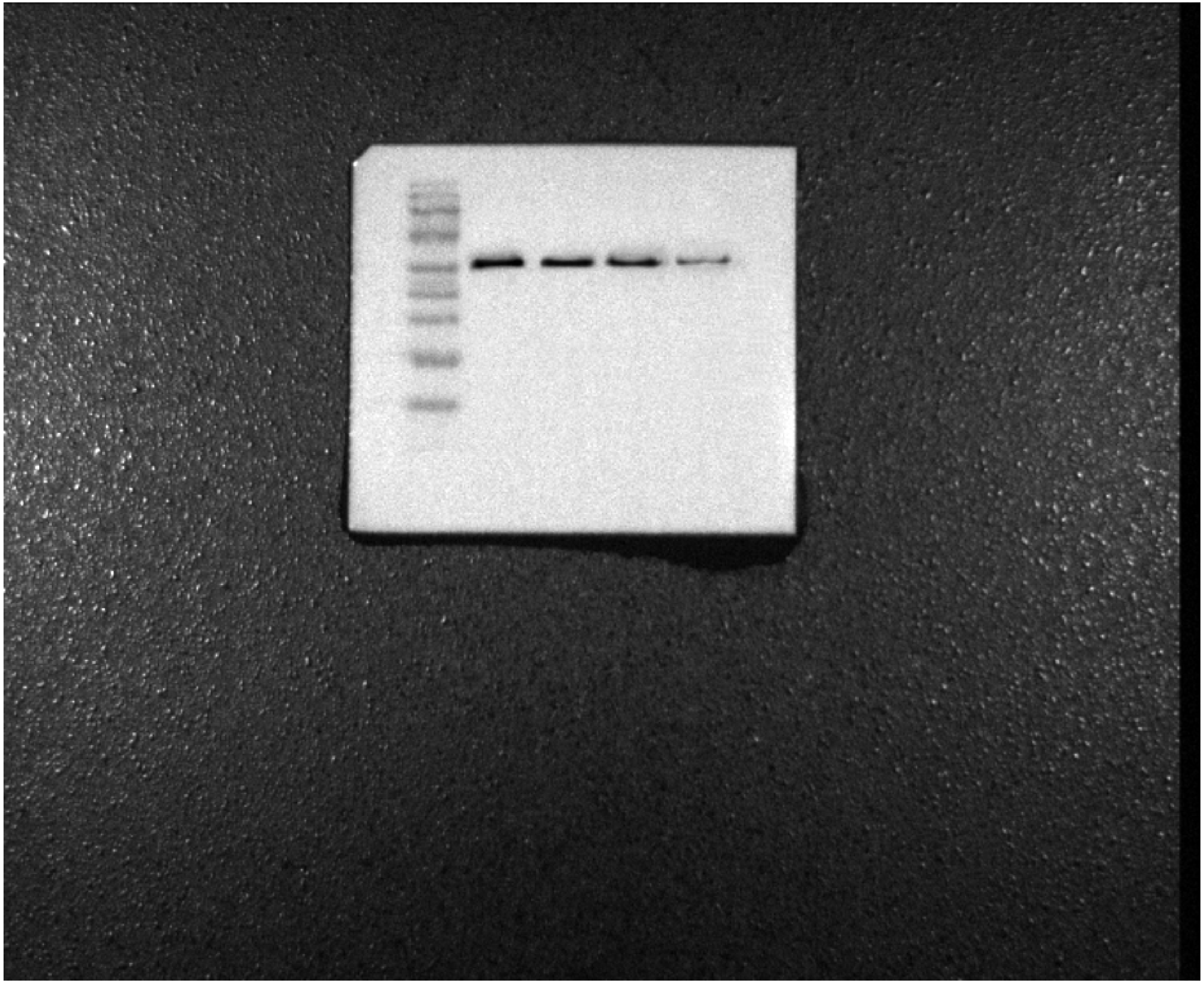

Figure 4A p-AKT (2)

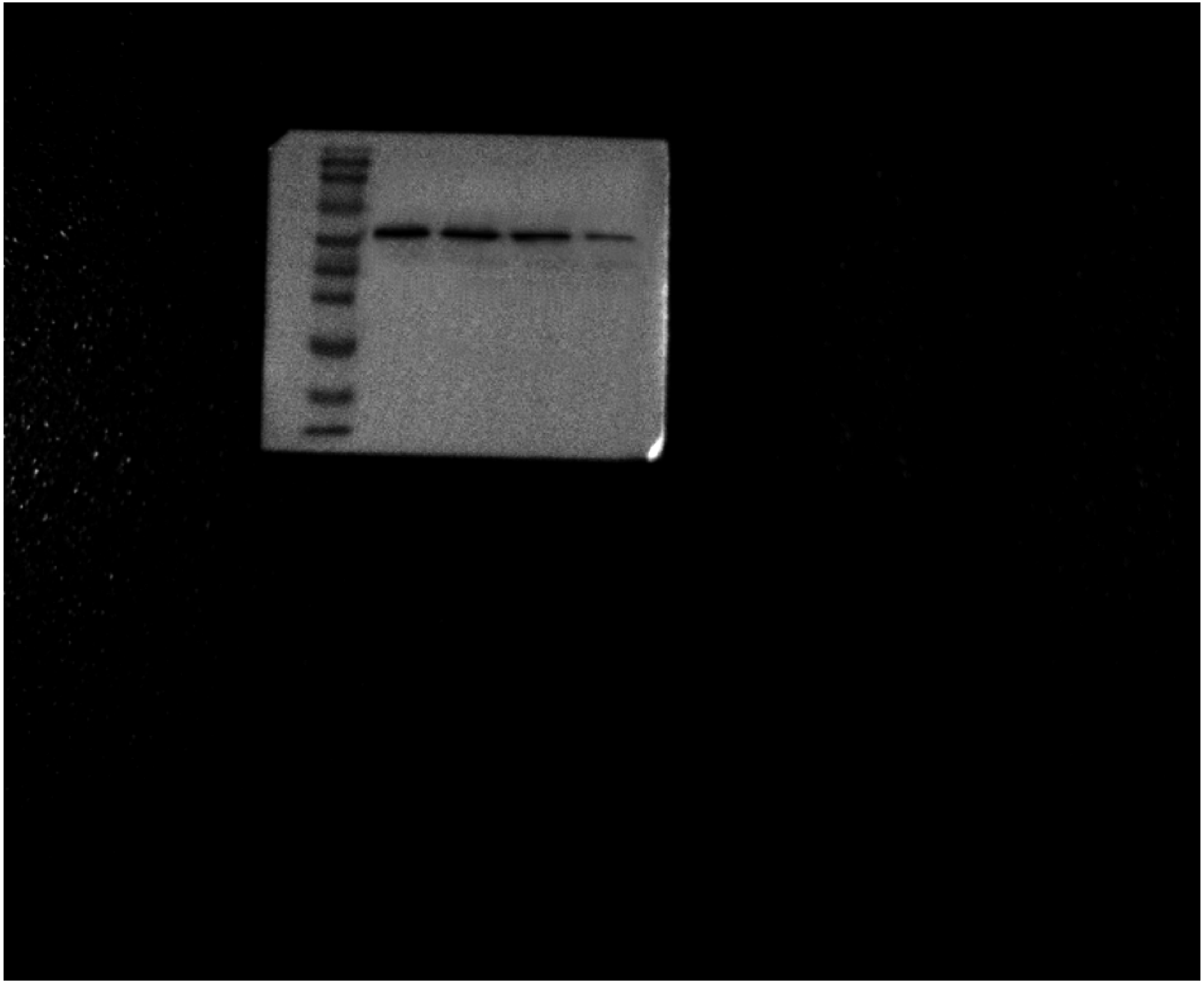

Figure 4A p-AKT (3)

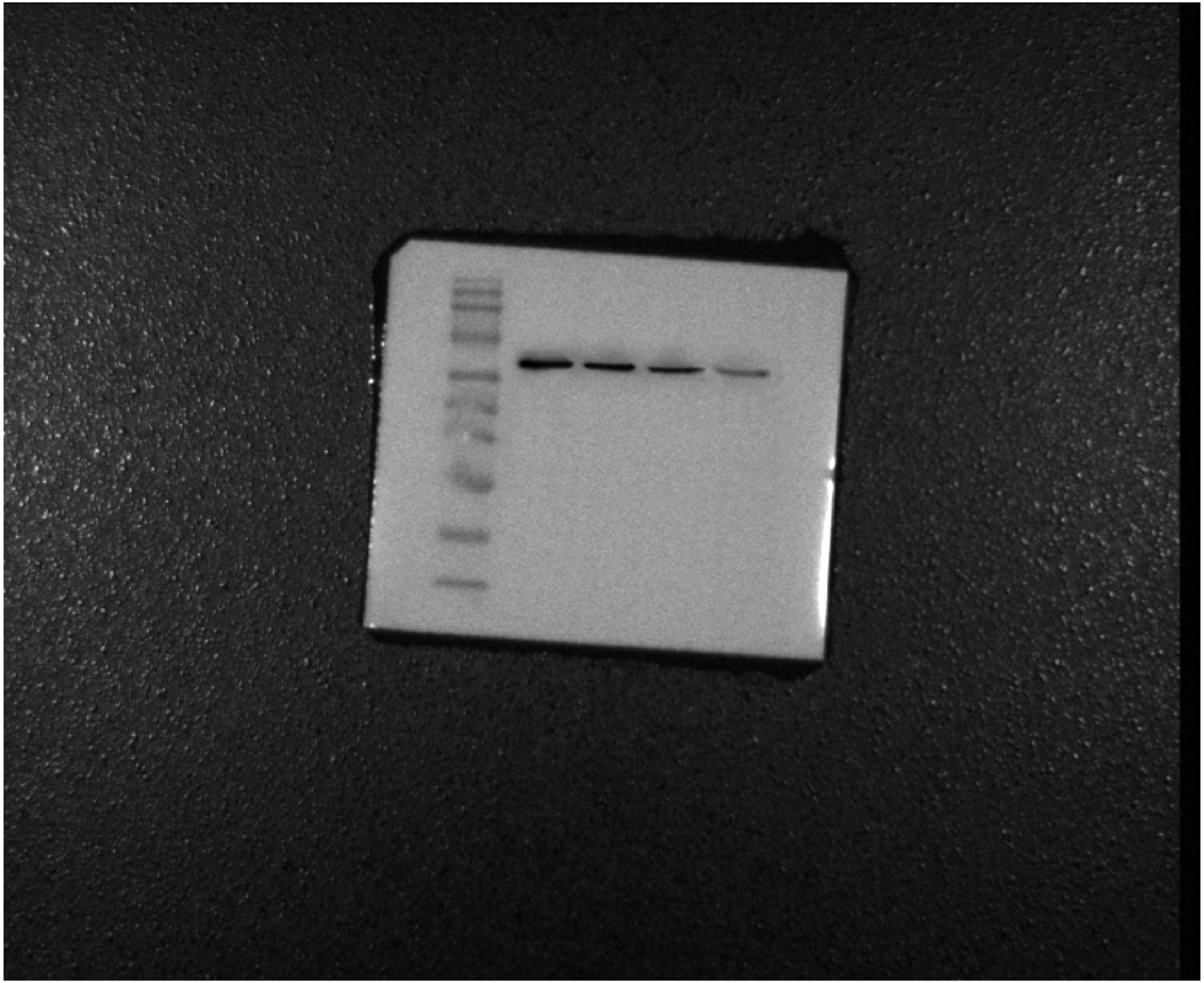

Figure 4A AKT (1)

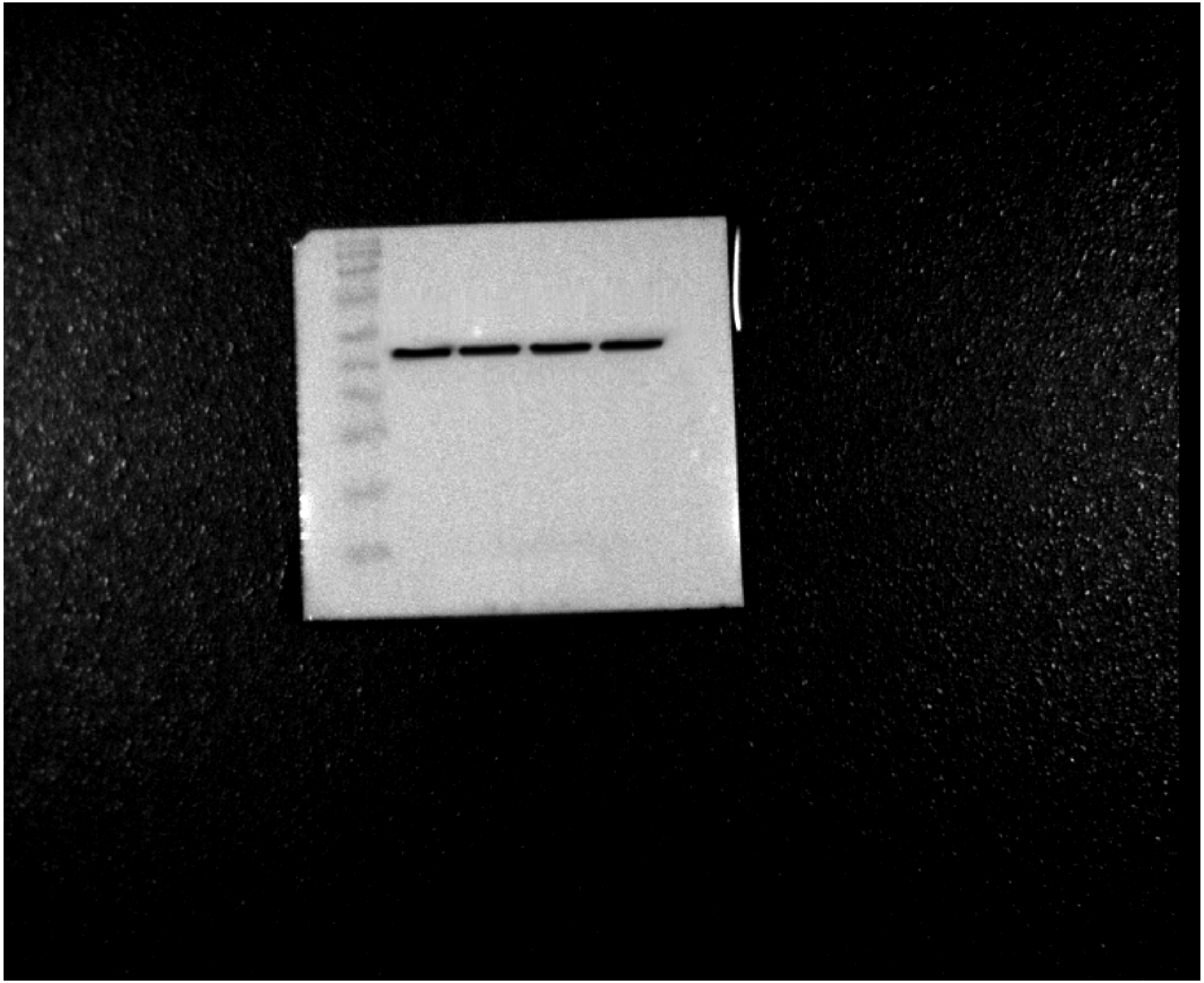

Figure 4A AKT (2)

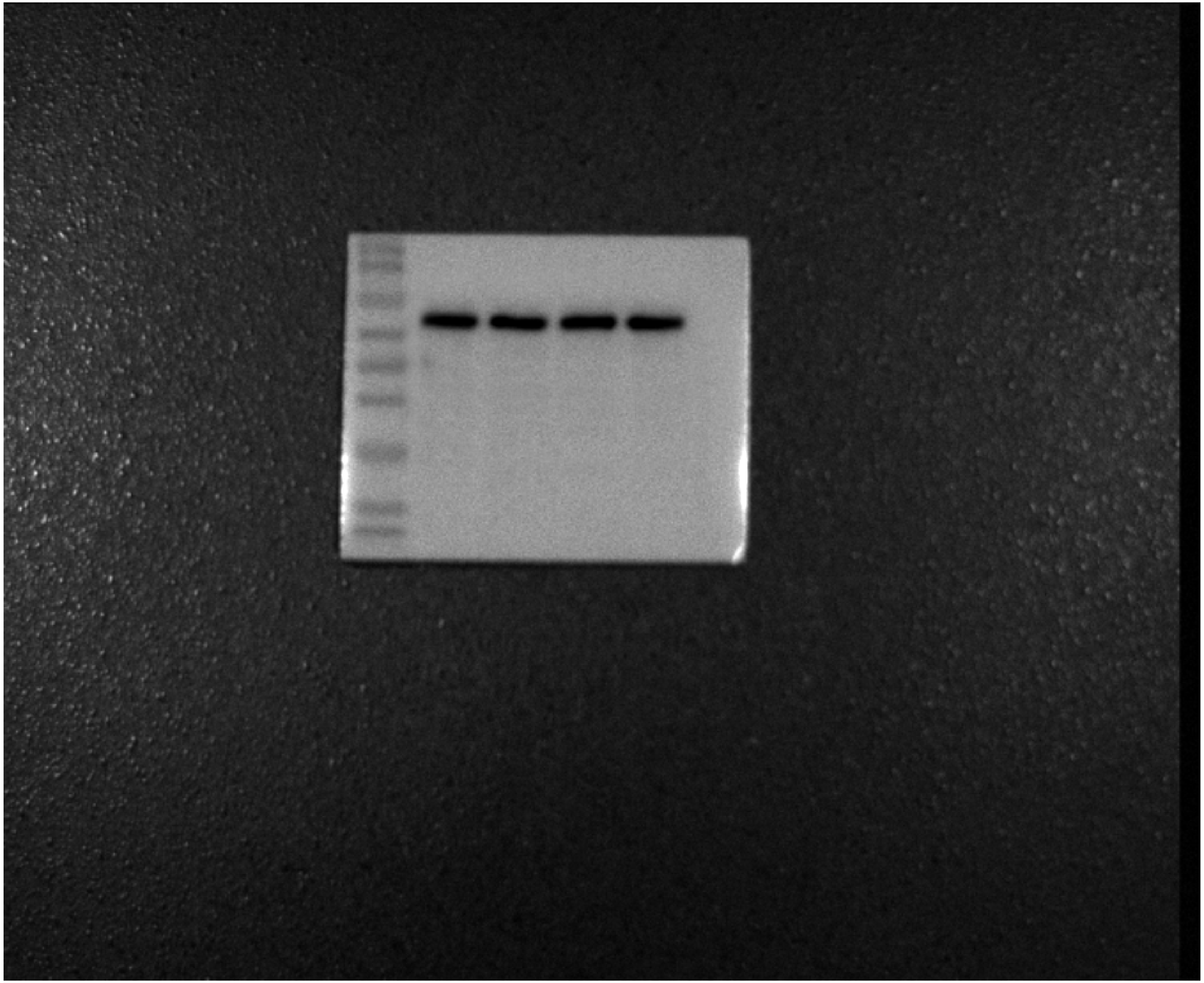

Figure 4A AKT (3)

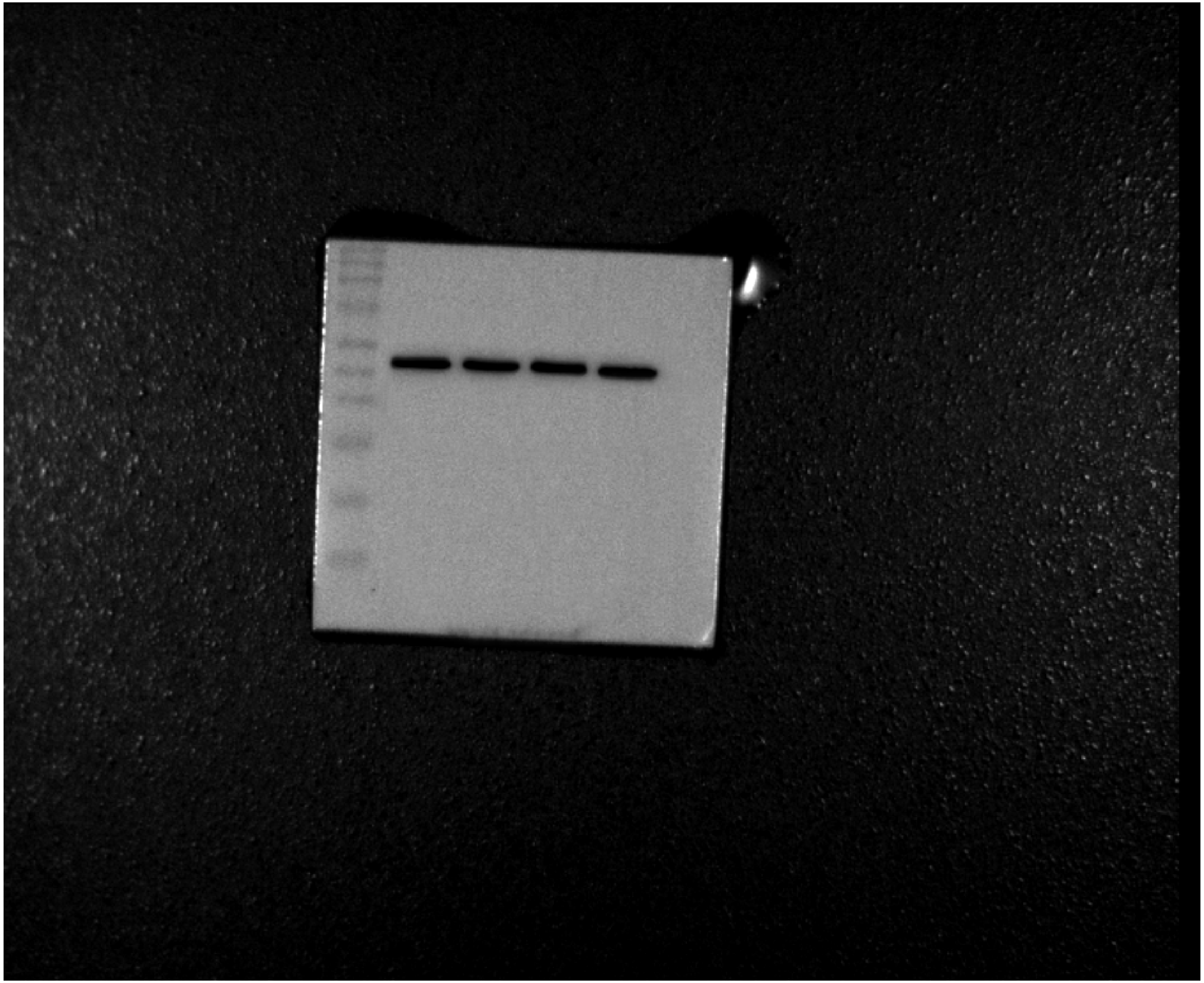

Figure 4A p-mTOR (1)

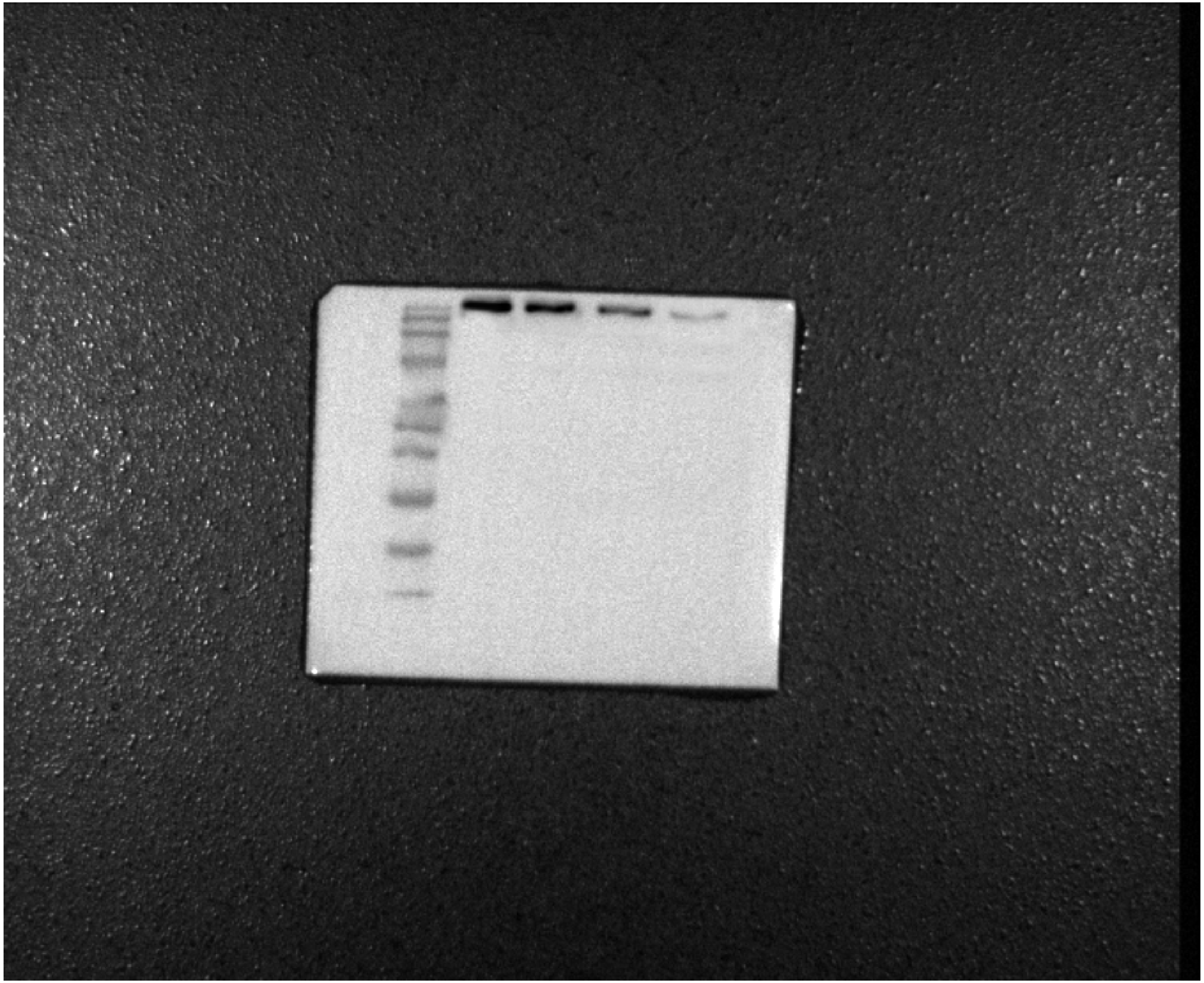

Figure 4A p-mTOR (2)

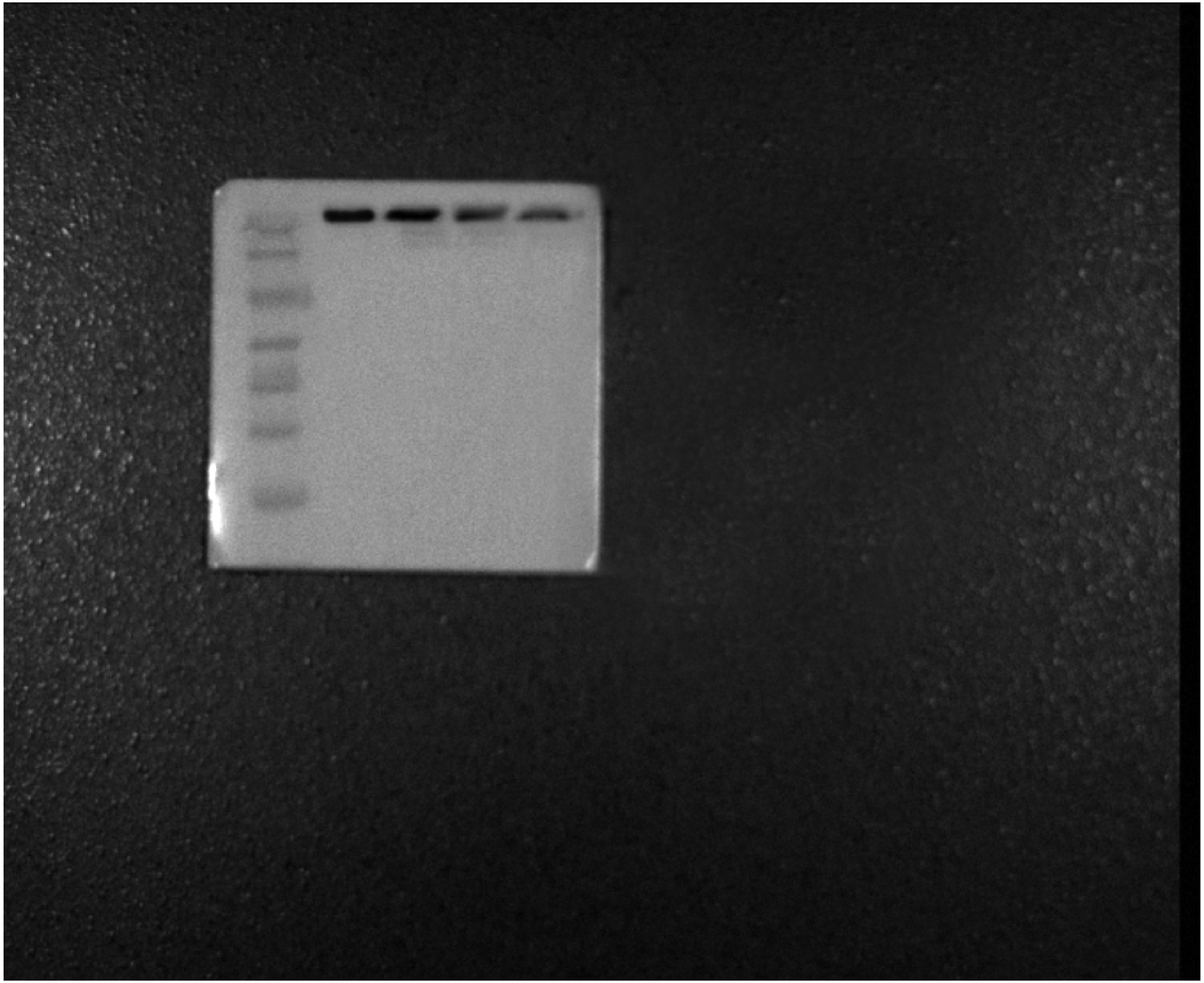

Figure 4A p-mTOR (3)

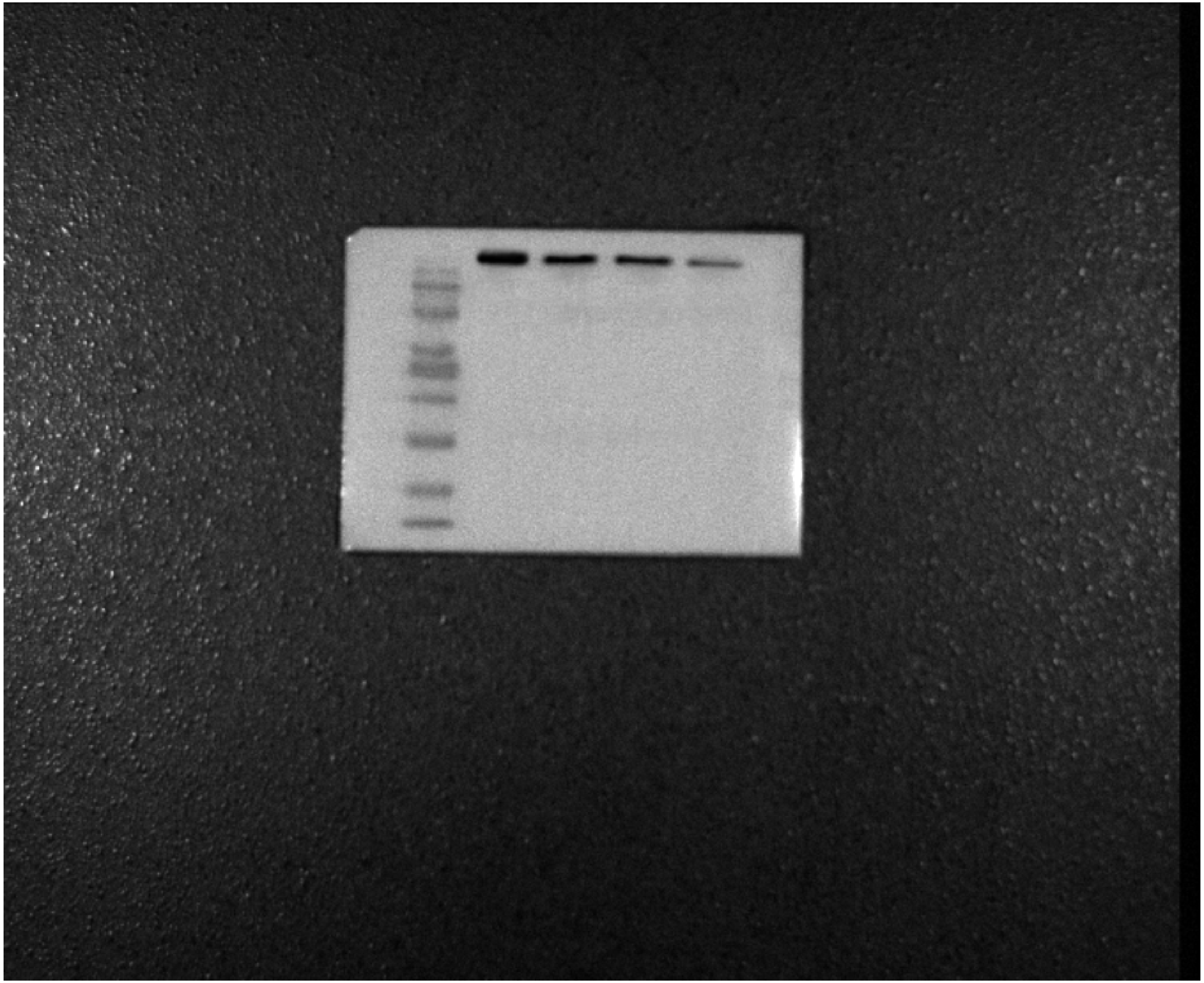

Figure 4A mTOR (1)

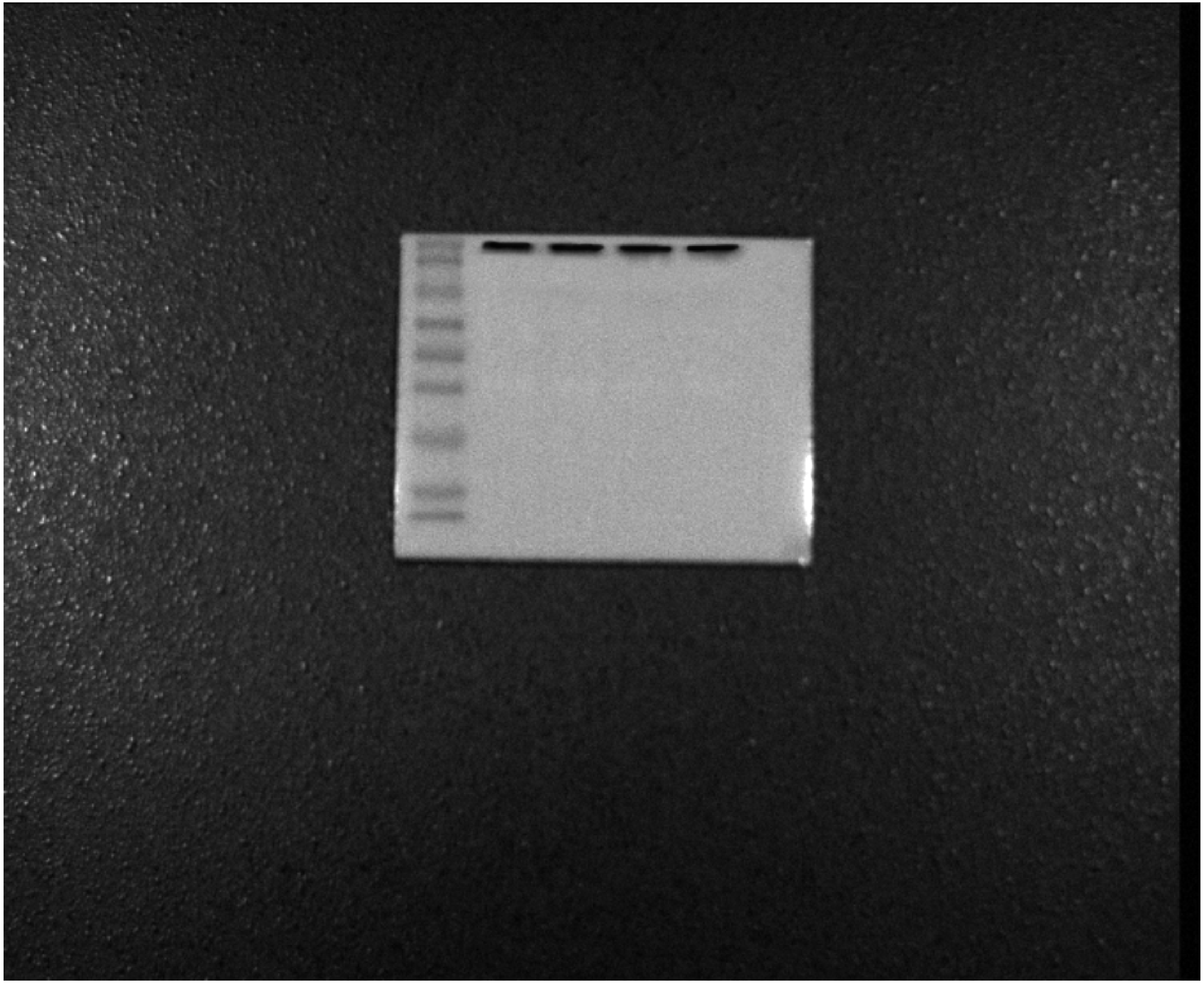

Figure 4A mTOR (2)

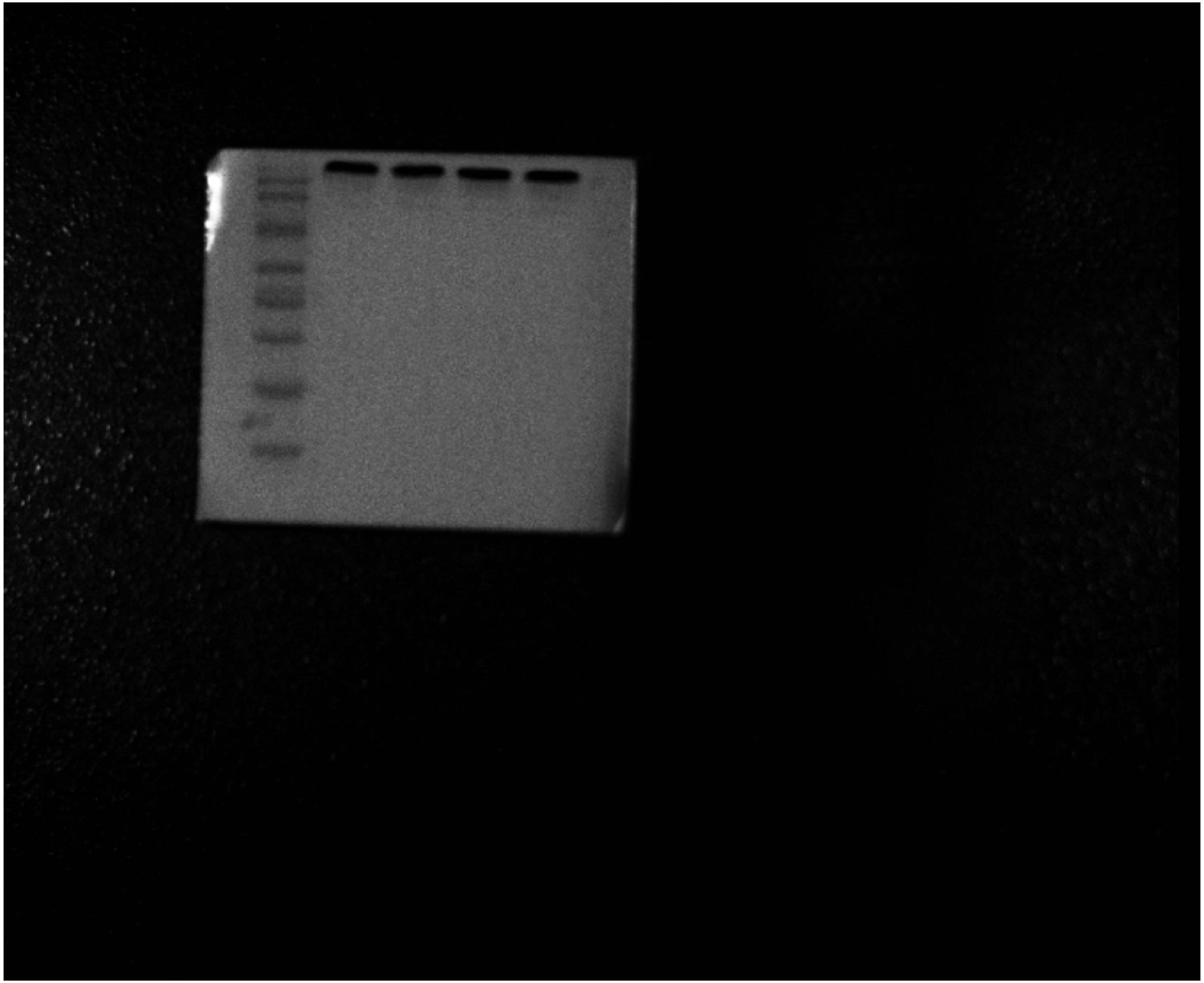

Figure 4A mTOR (3)

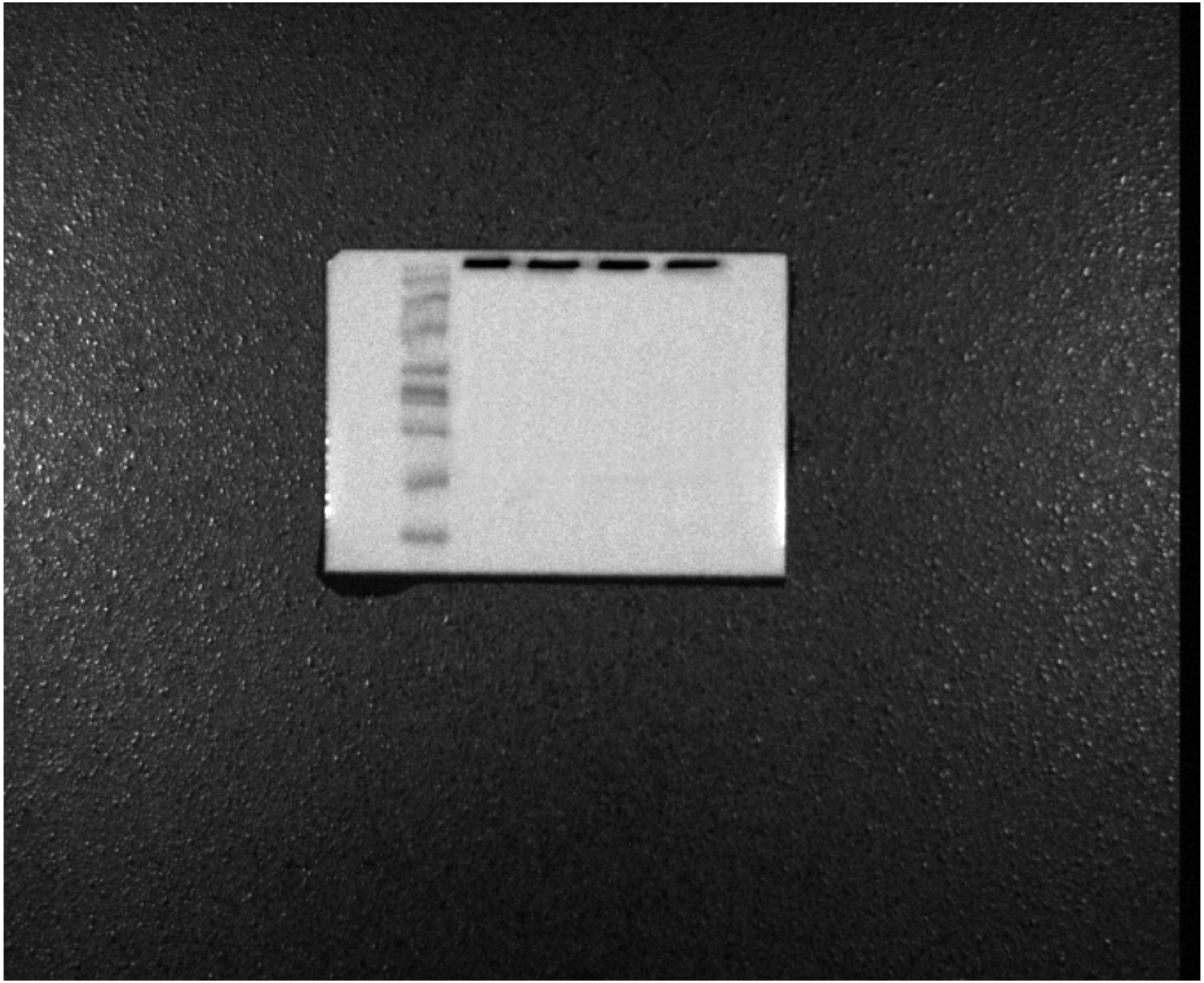

Figure 4A GAPDH (1)

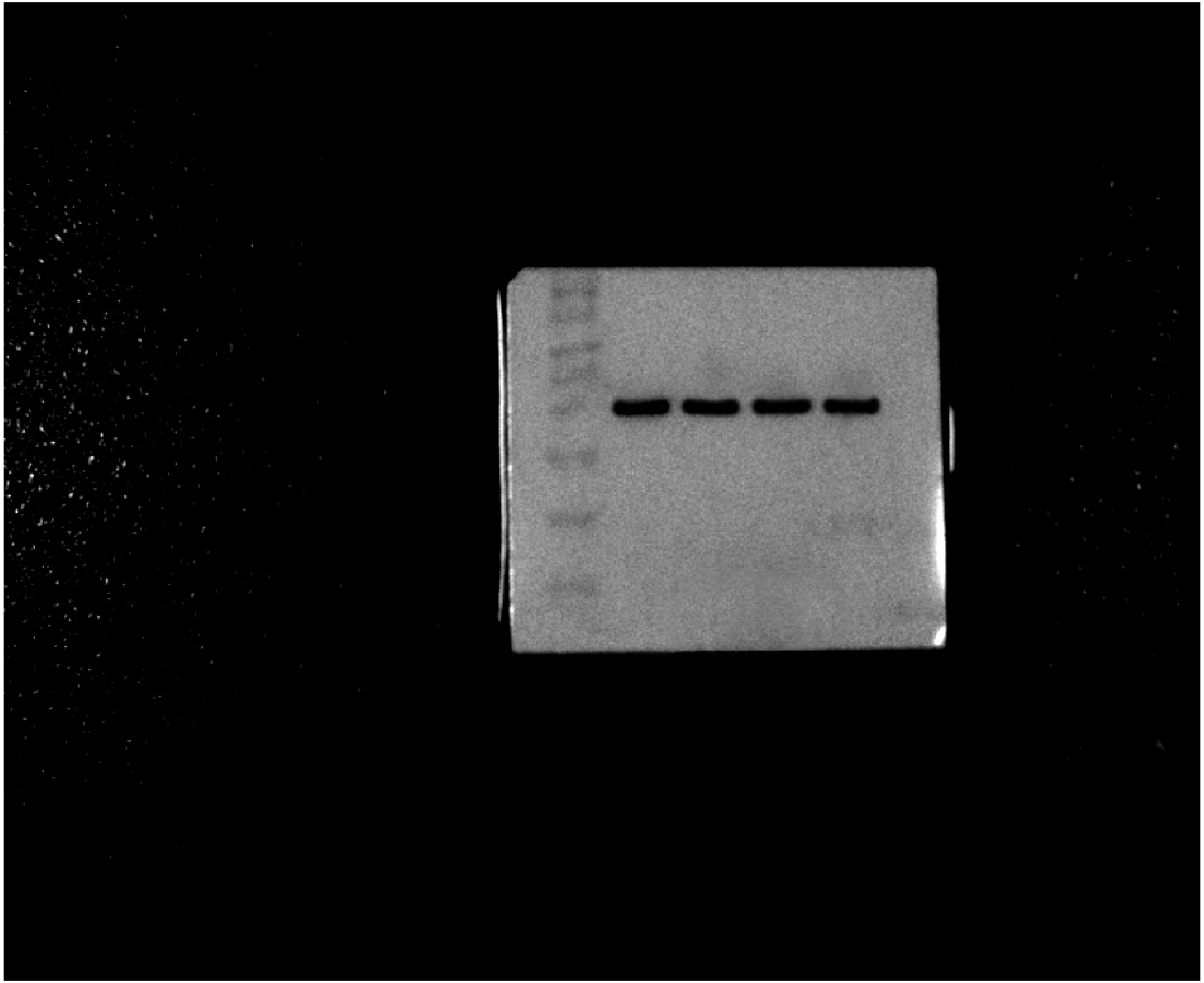

Figure 4A GAPDH (2)

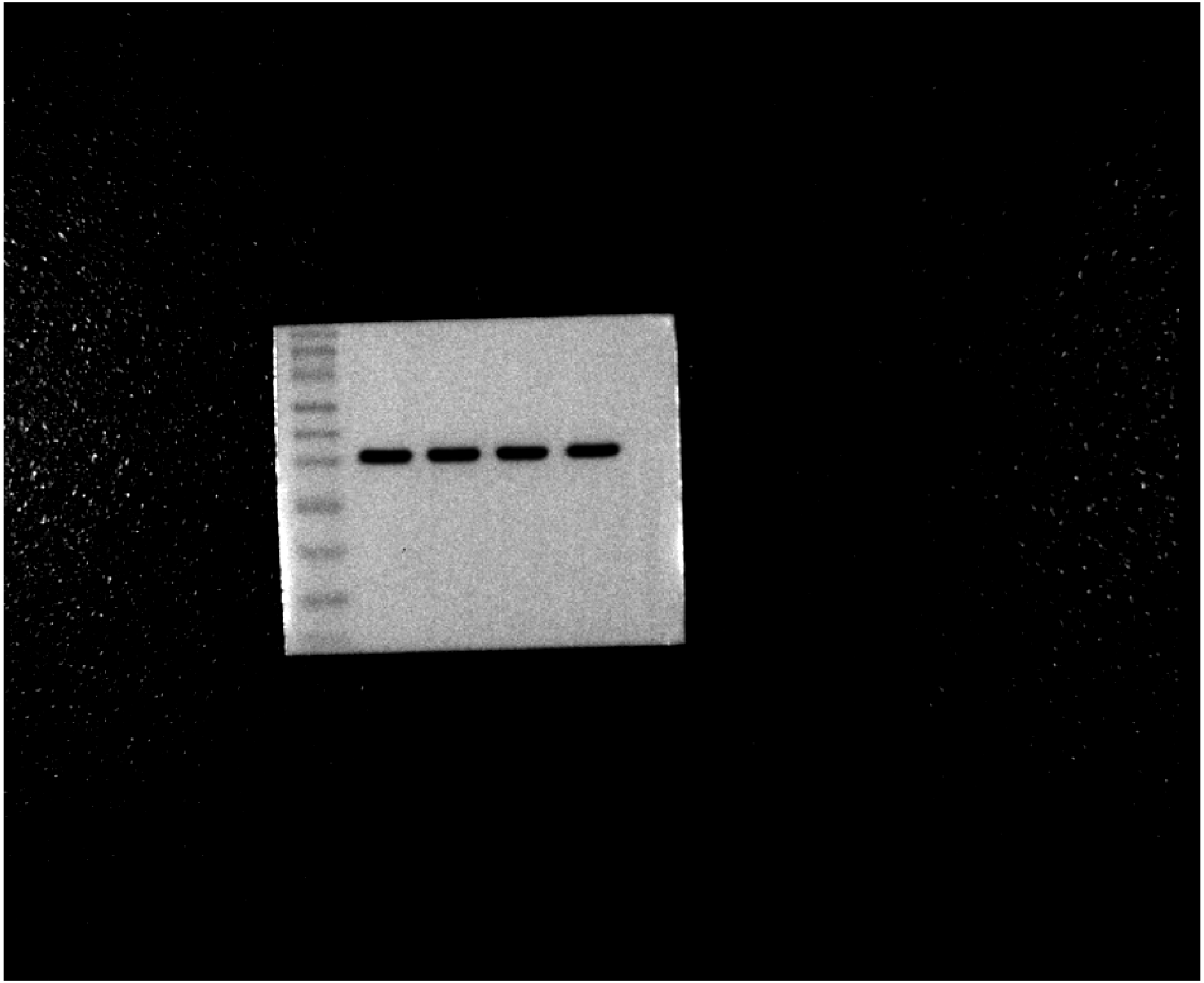

Figure 4A GAPDH (3)

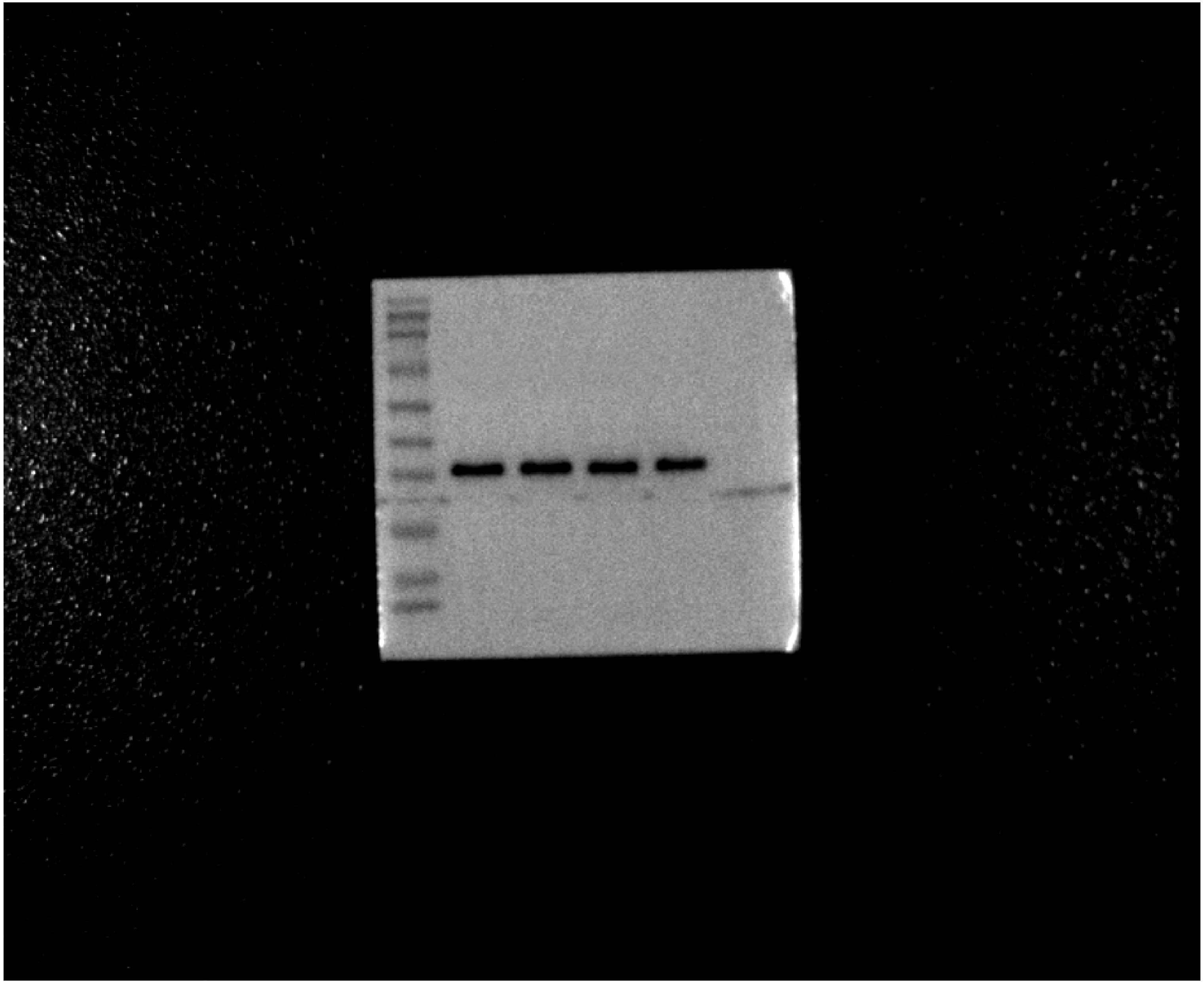

Figure 5A p-PI3K (1)

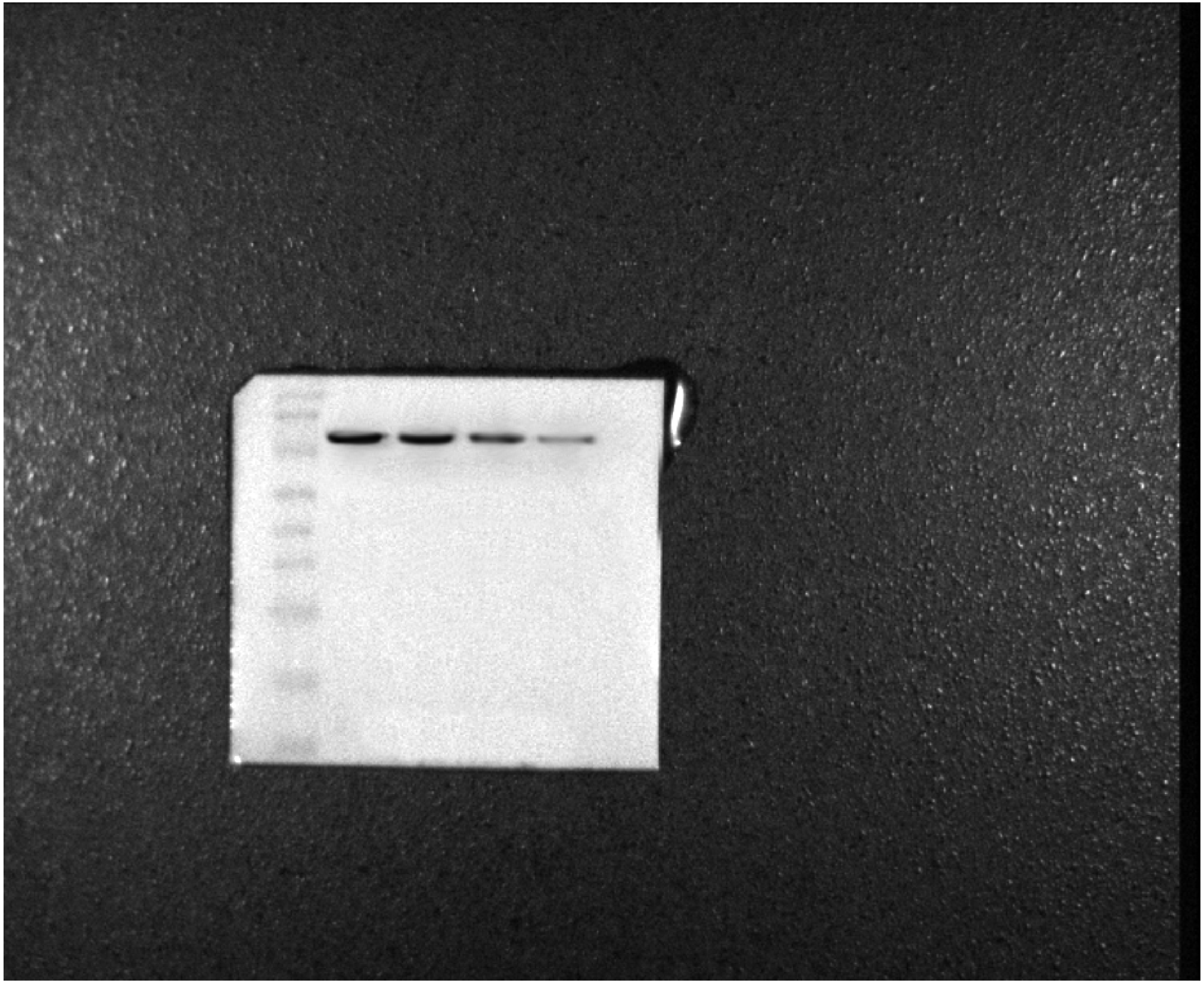

Figure 5A p-PI3K (2)

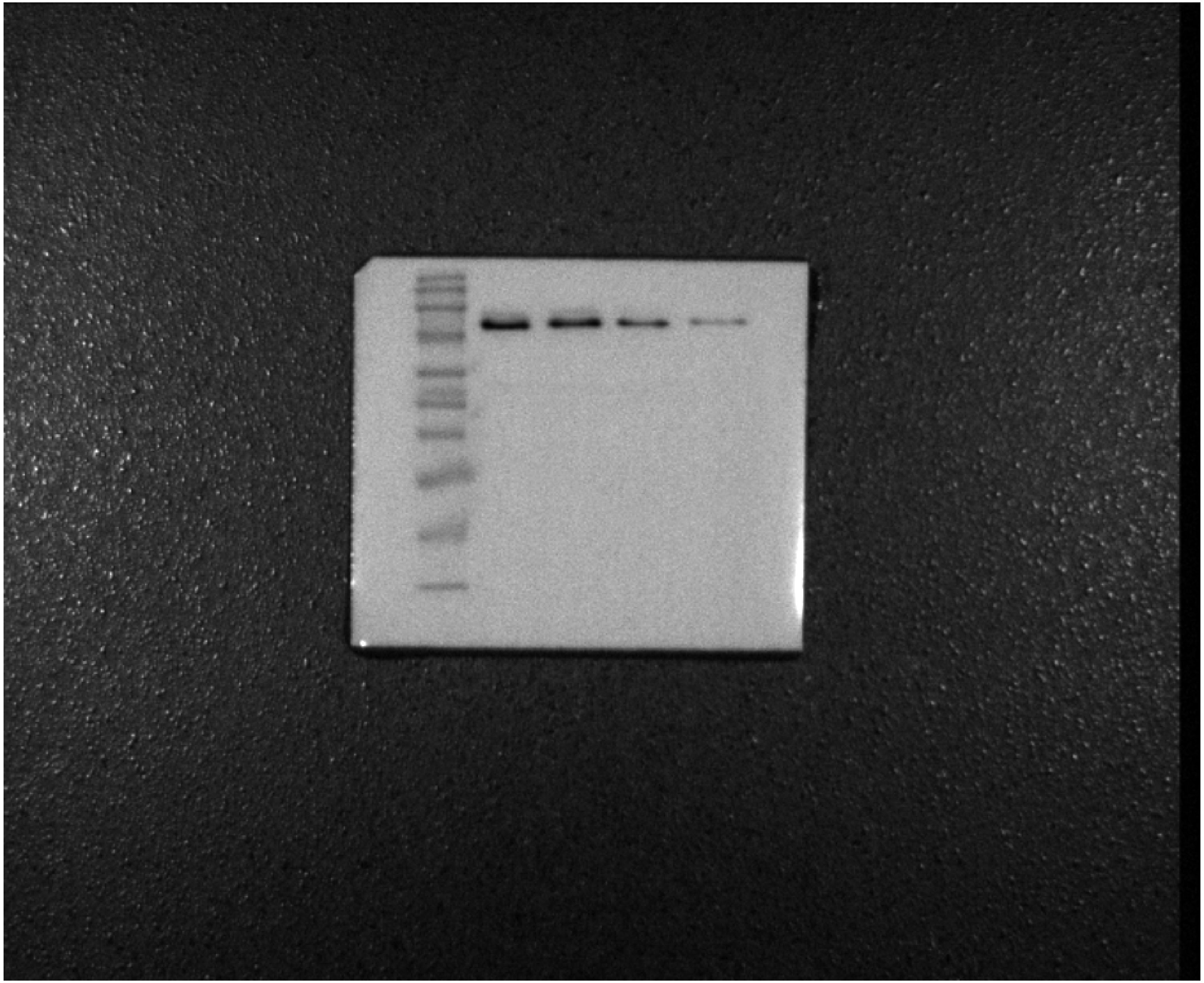

Figure 5A p-PI3K (3)

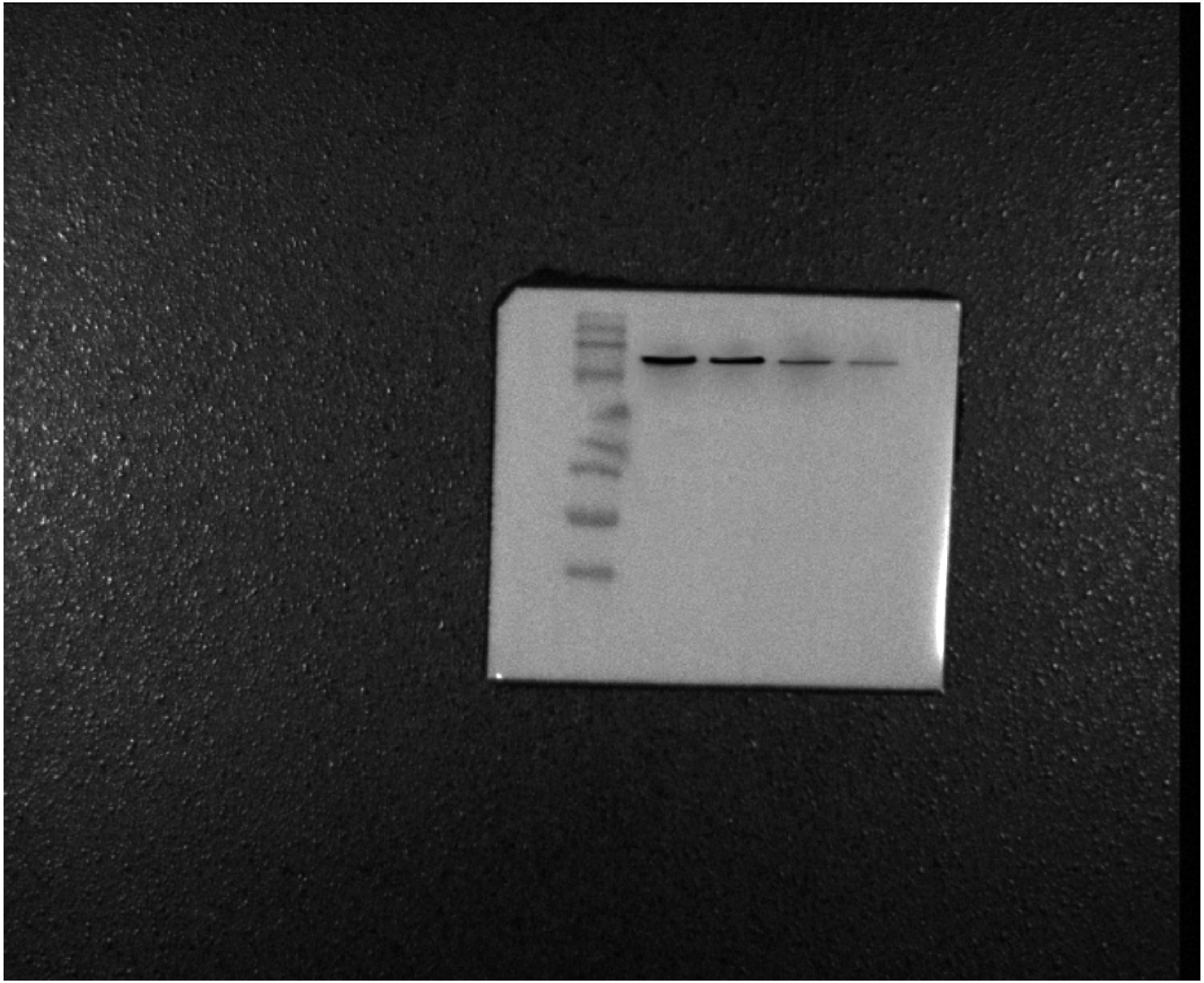

Figure 5A PI3K (1)

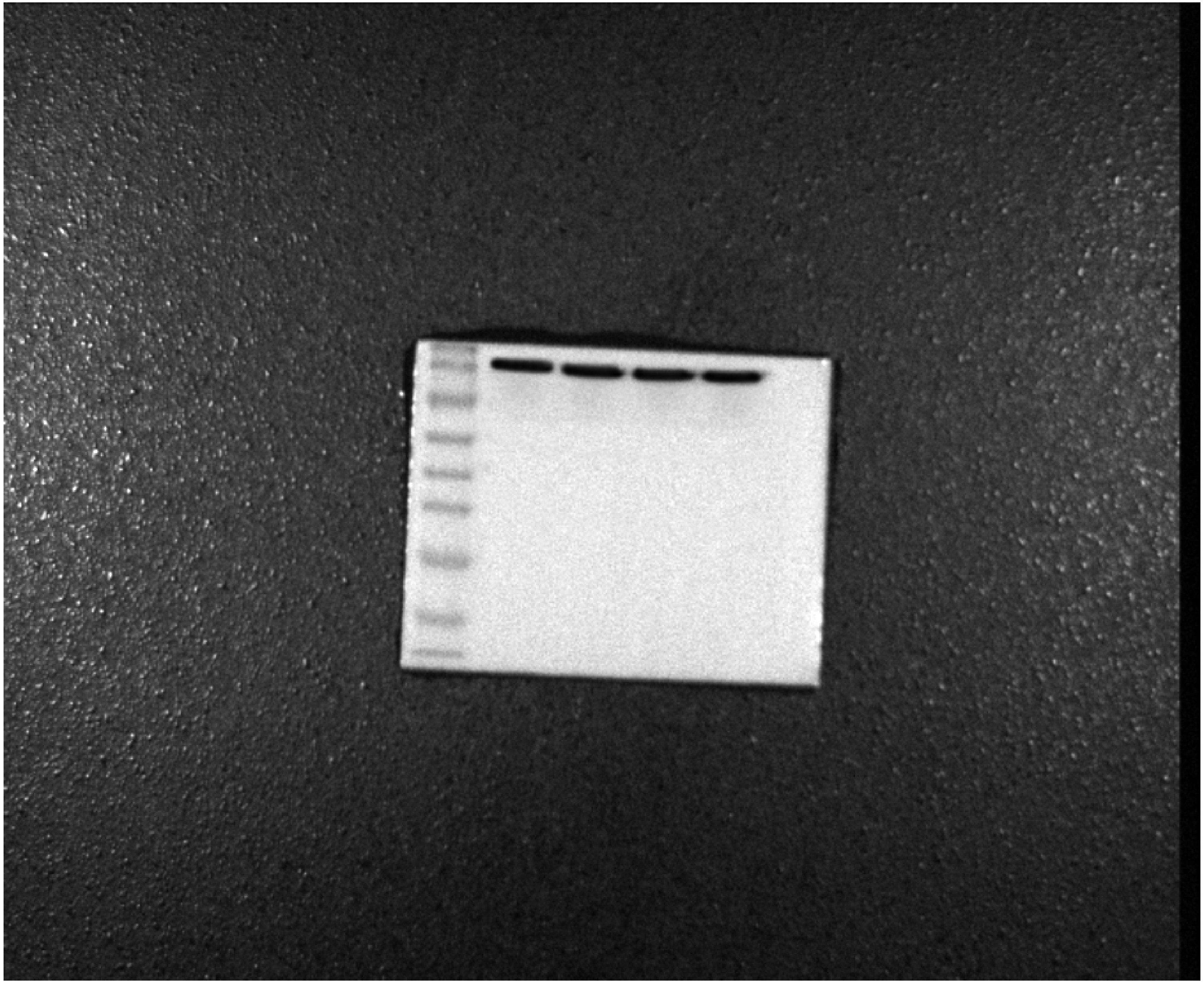

Figure 5A PI3K (2)

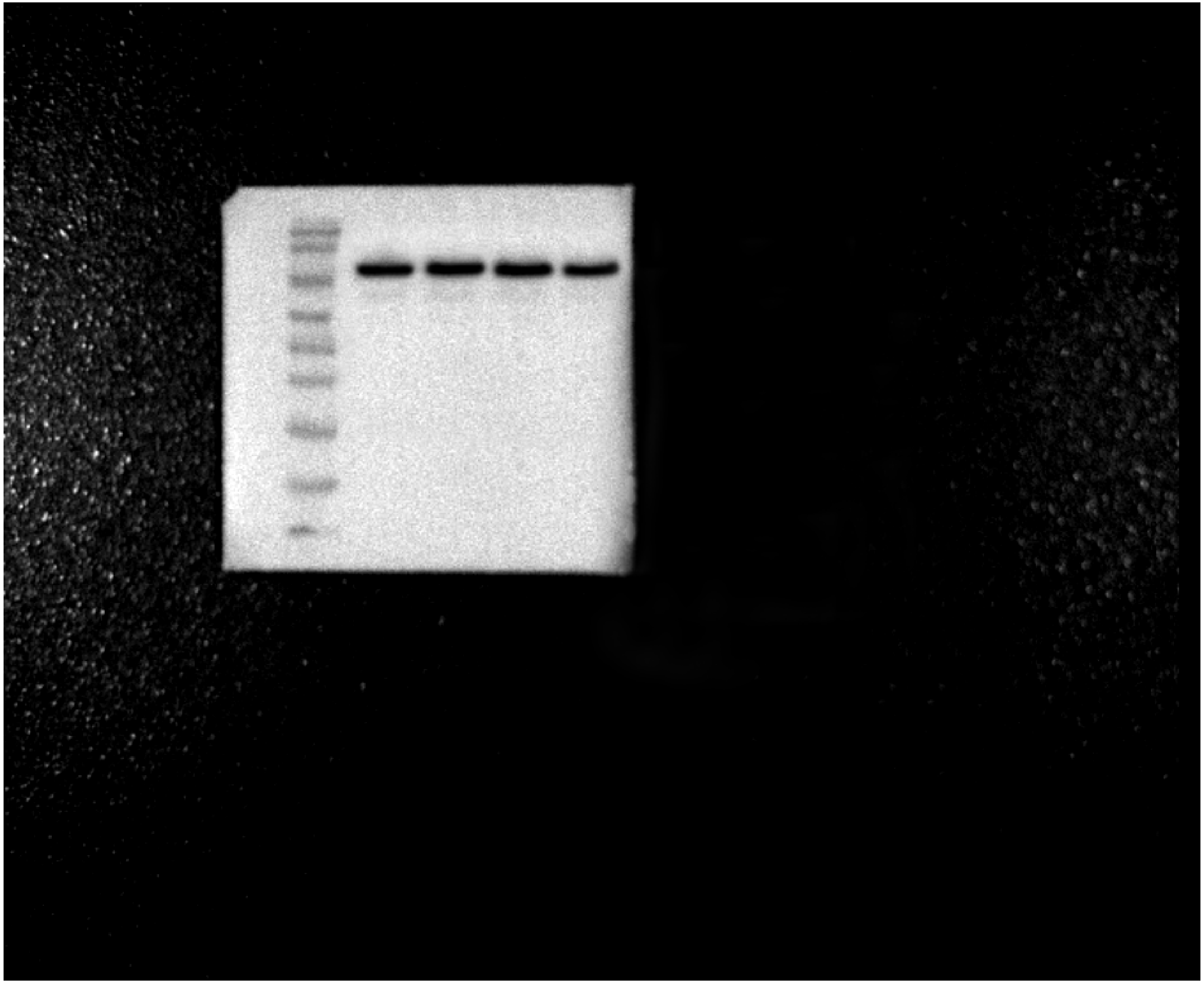

Figure 5A PI3K (3)

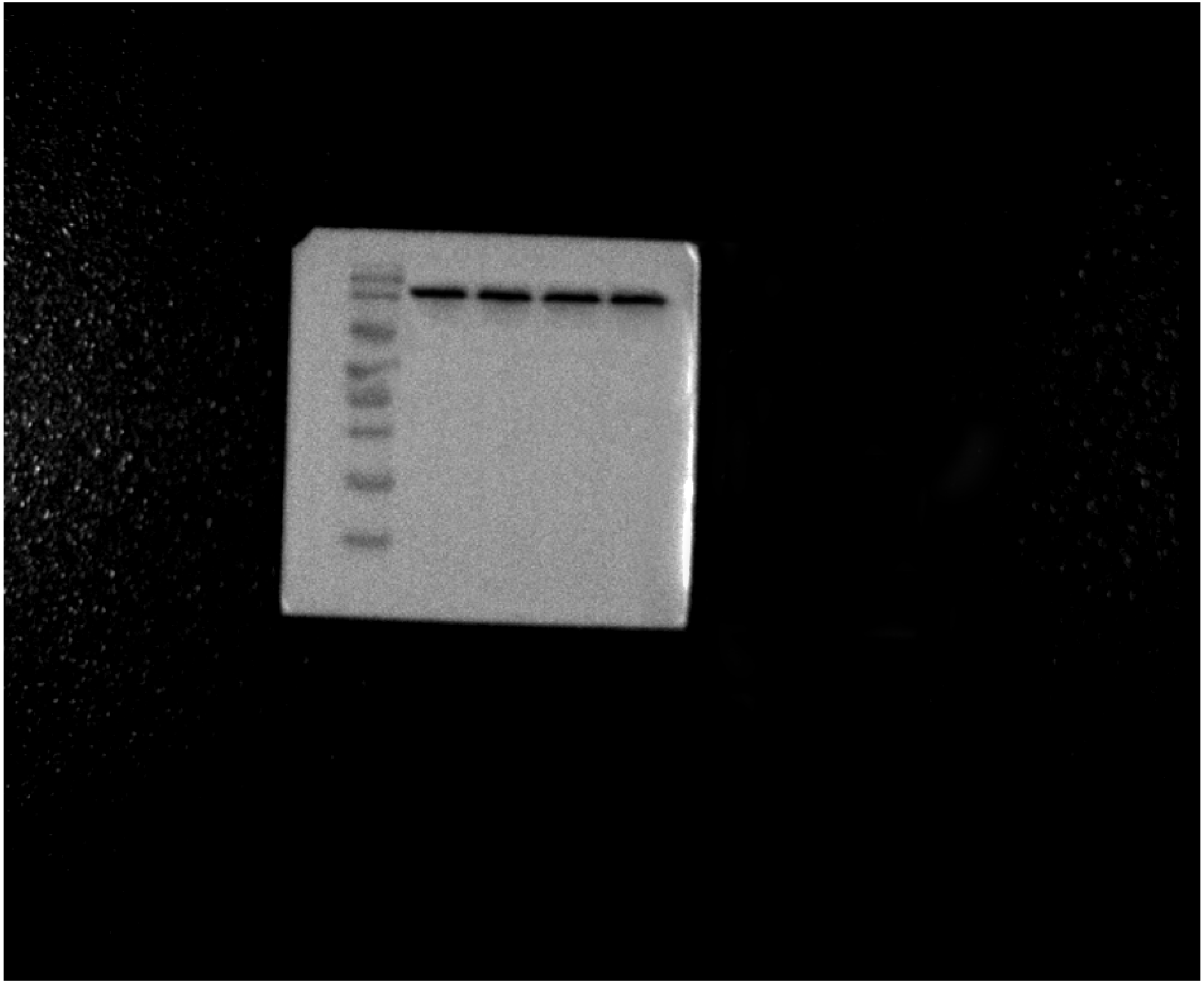

Figure 5A p-AKT (1)

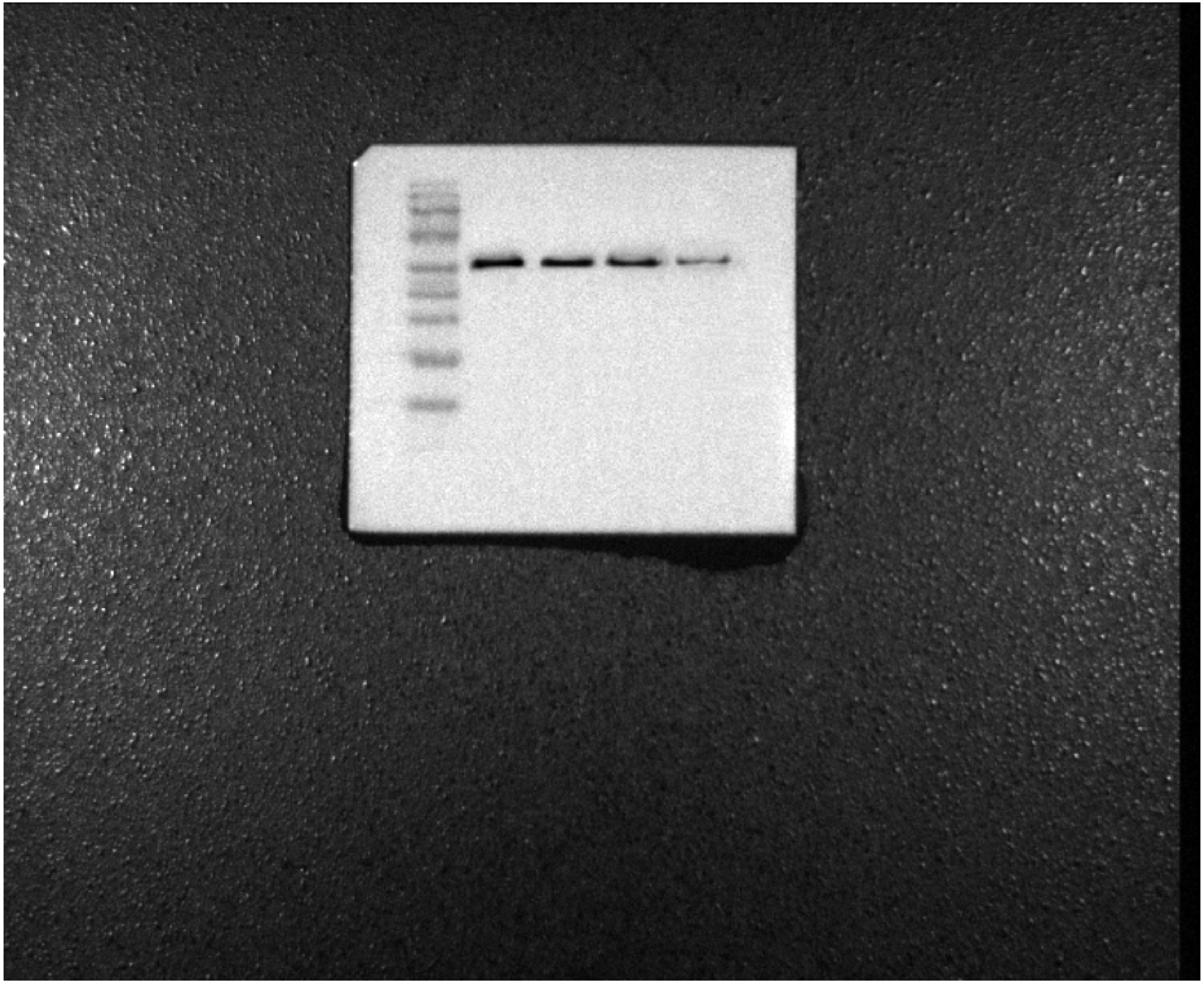

Figure 5A p-AKT (2)

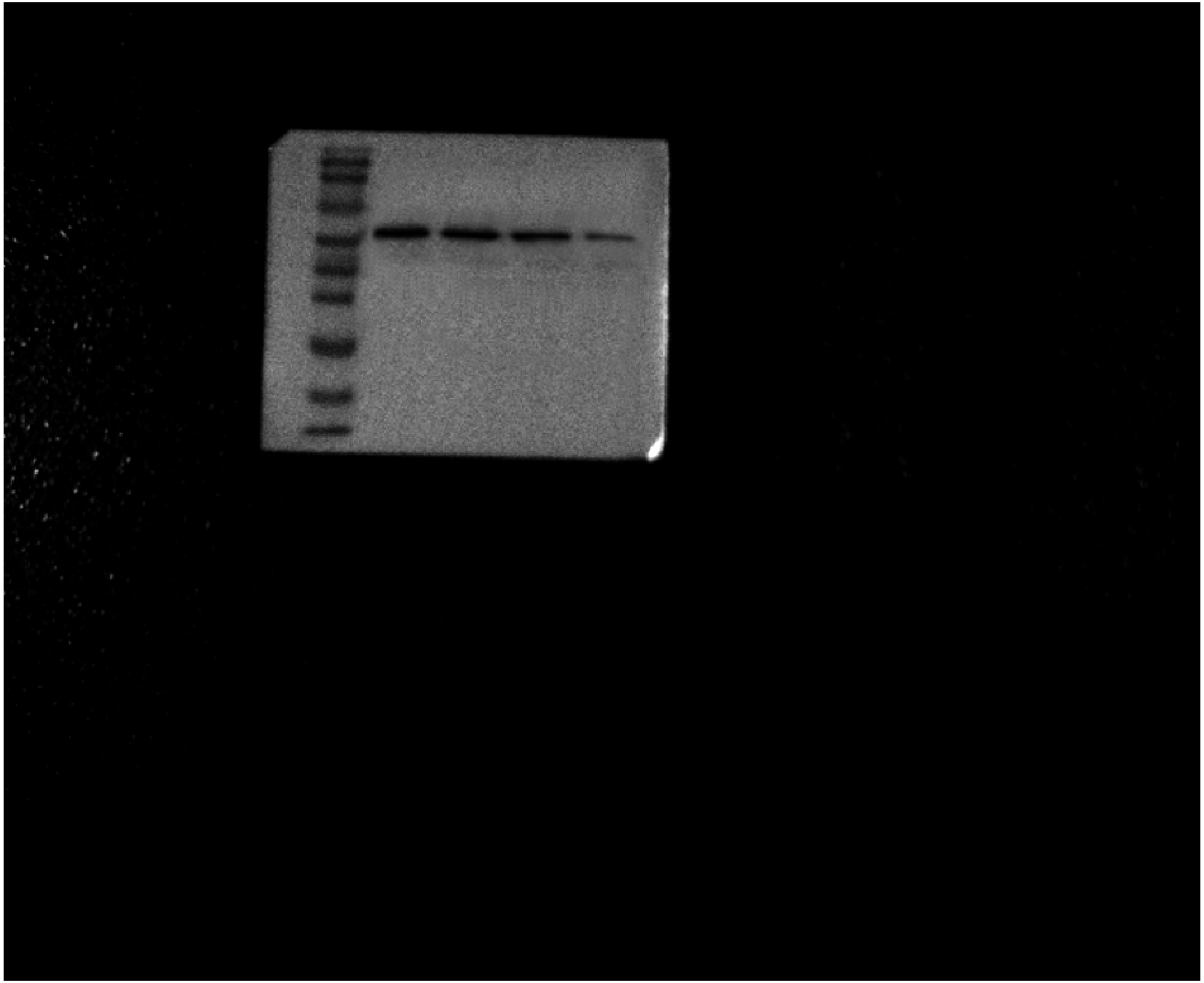

Figure 5A p-AKT (3)

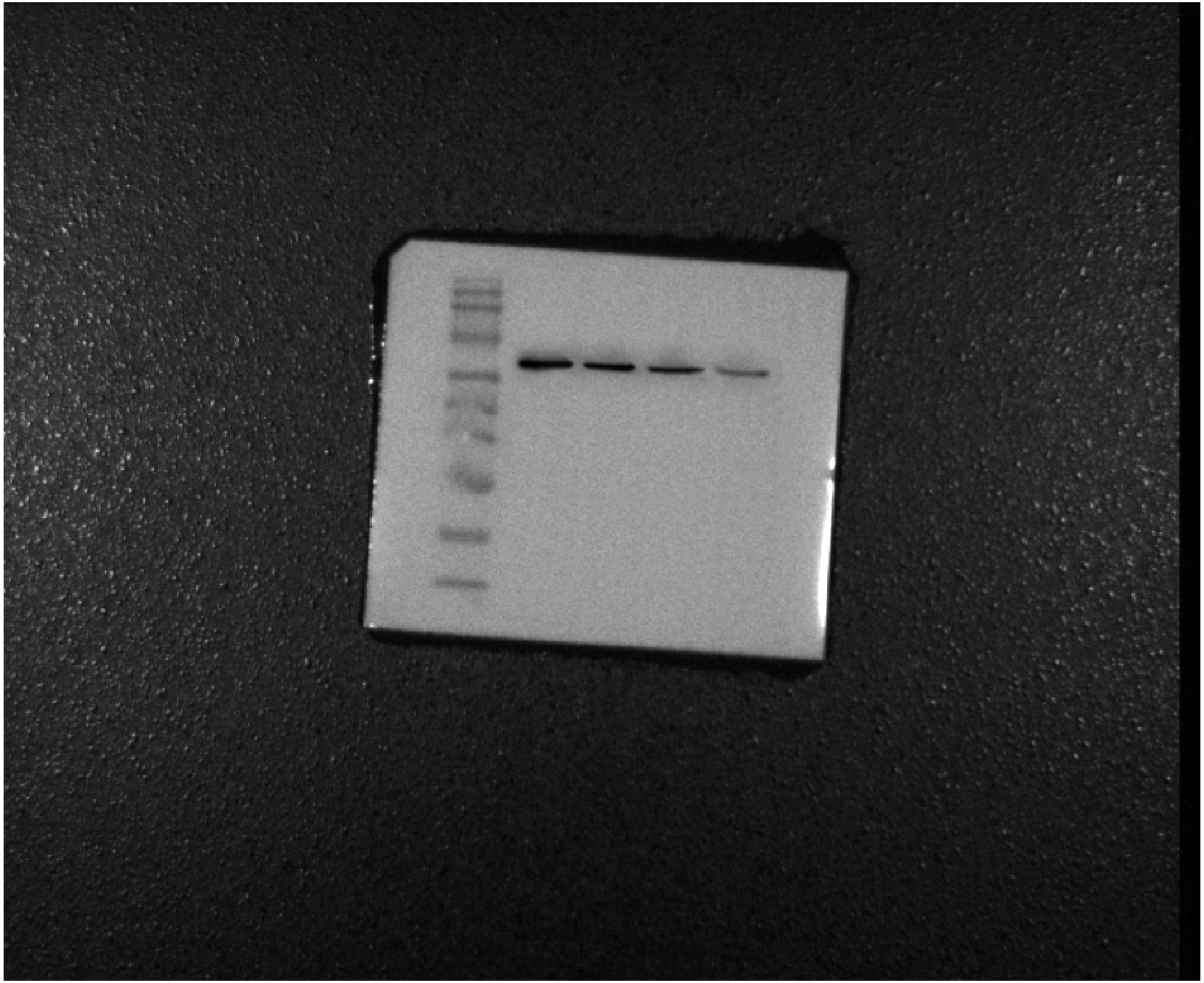

Figure 5A AKT (1)

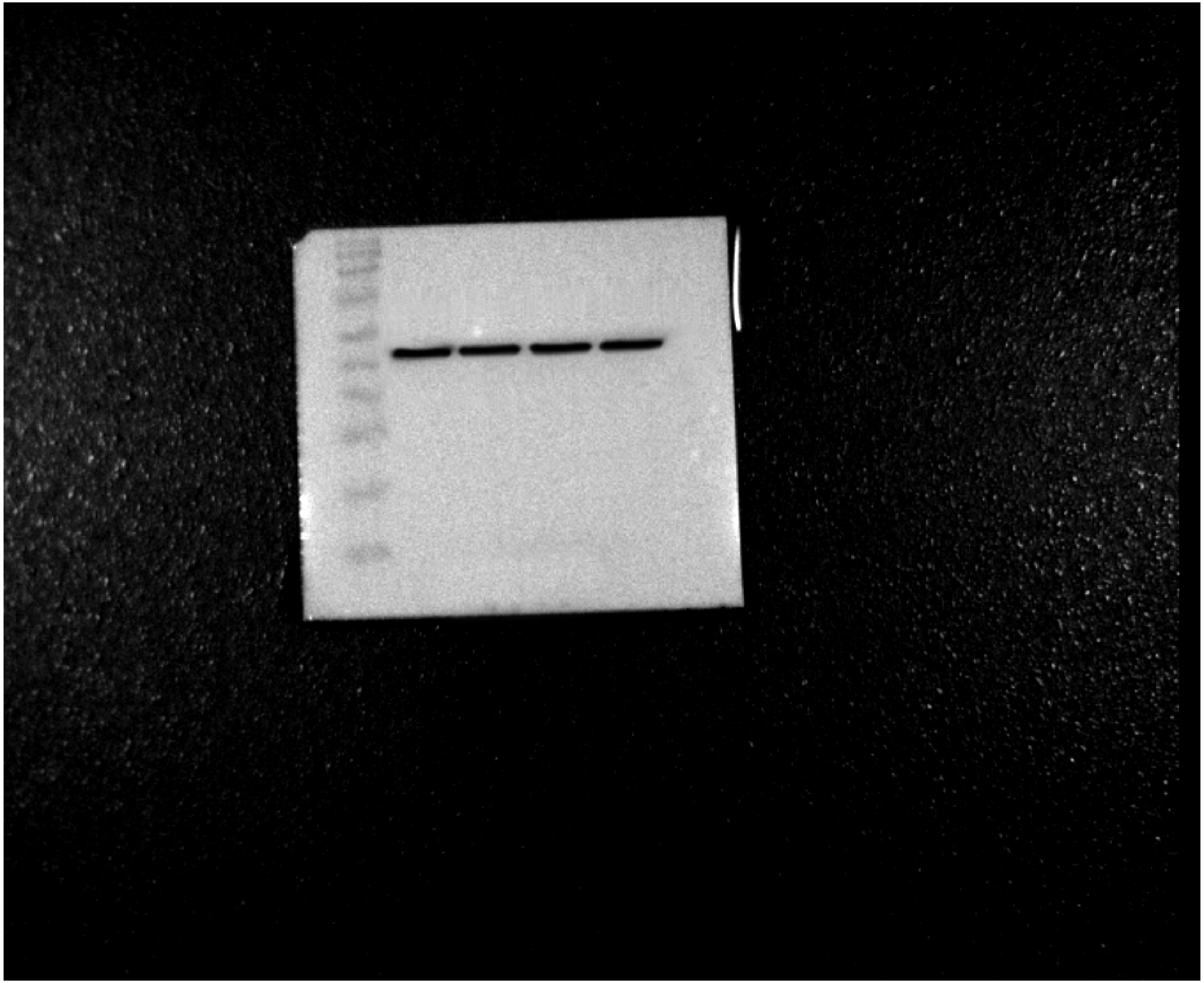

Figure 5A AKT (2)

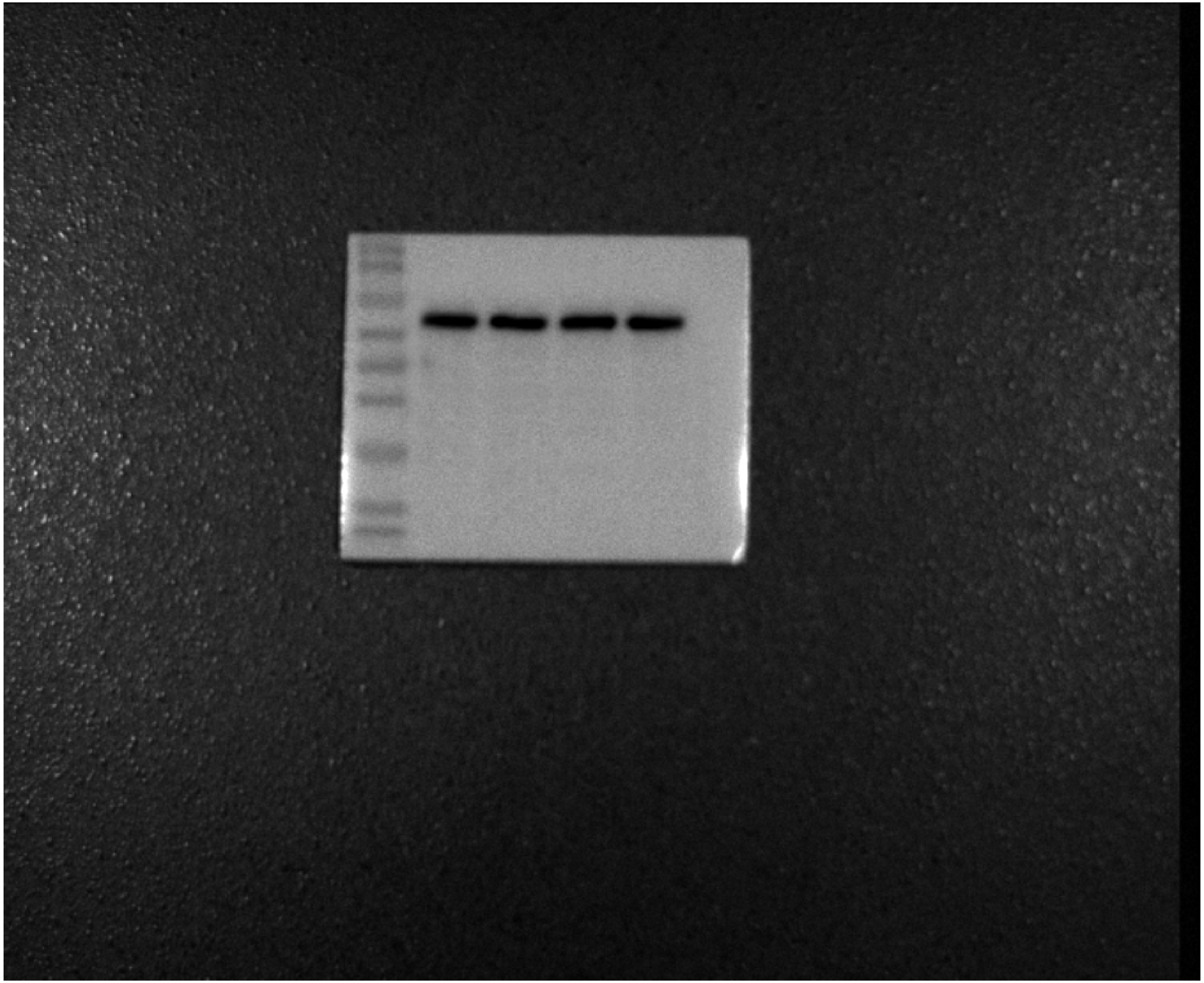

Figure 5A AKT (3)

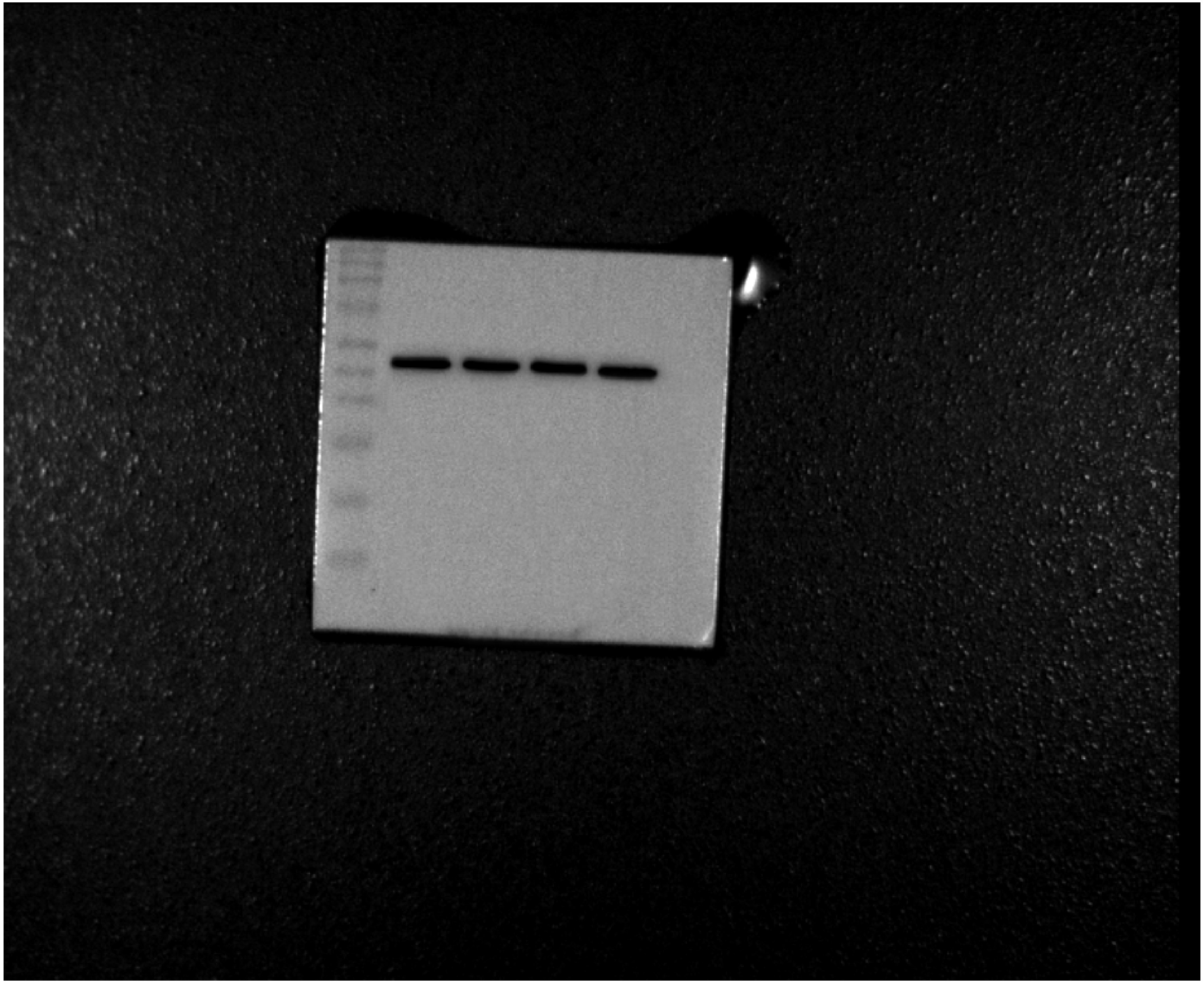

Figure 5A p-mTOR (1)

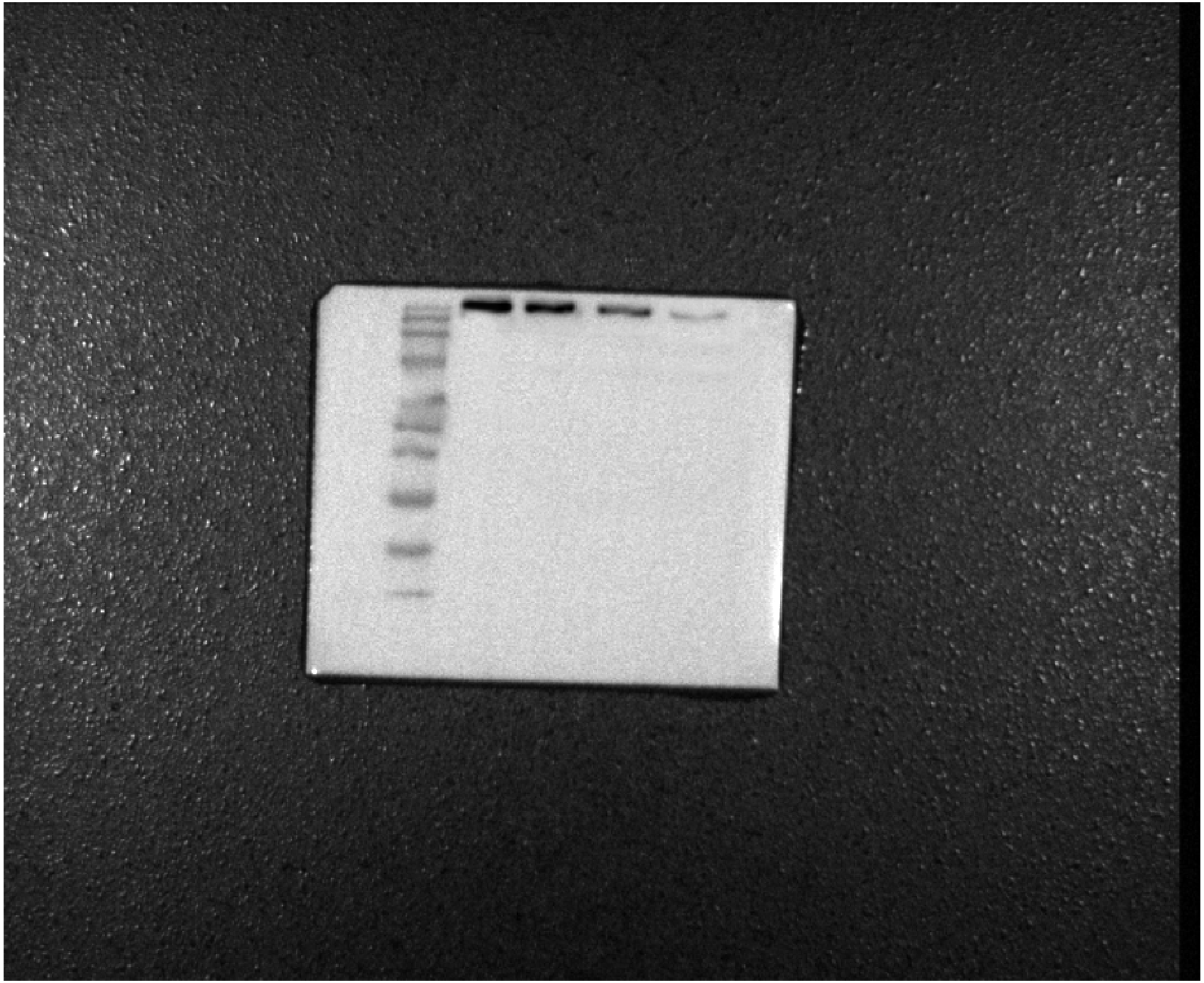

Figure 5A p-mTOR (2)

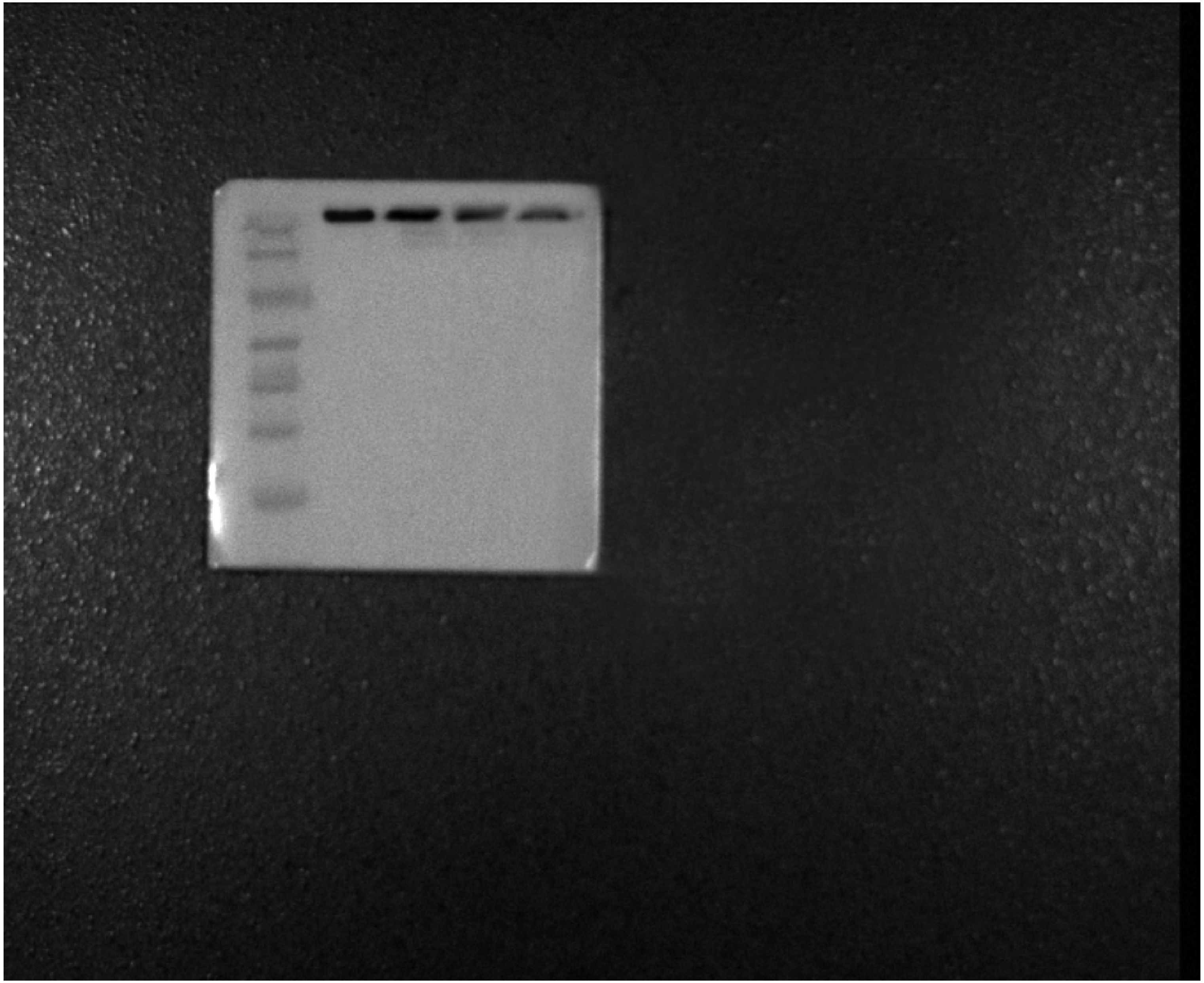

Figure 5A p-mTOR (3)

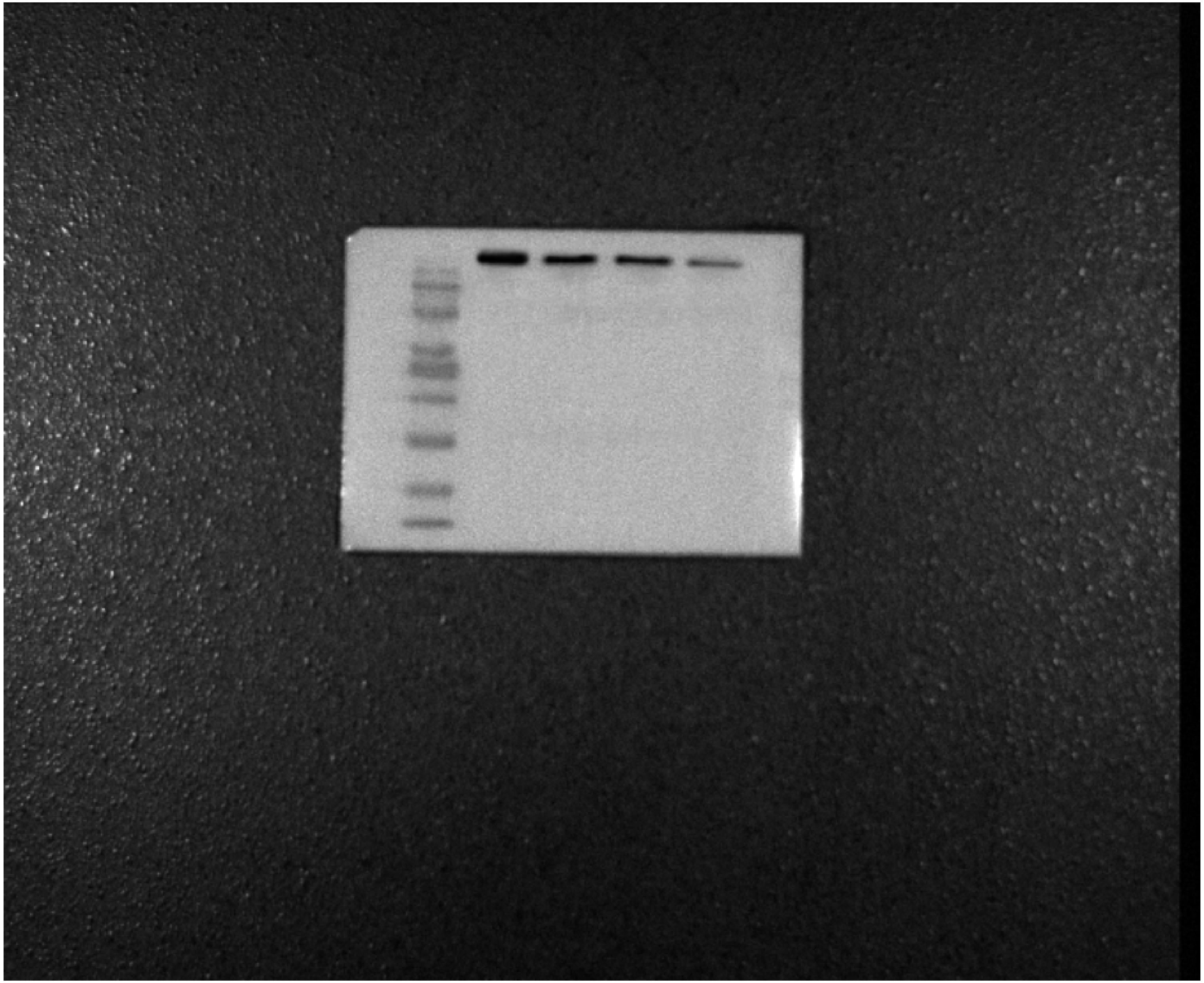

Figure 5A mTOR (1)

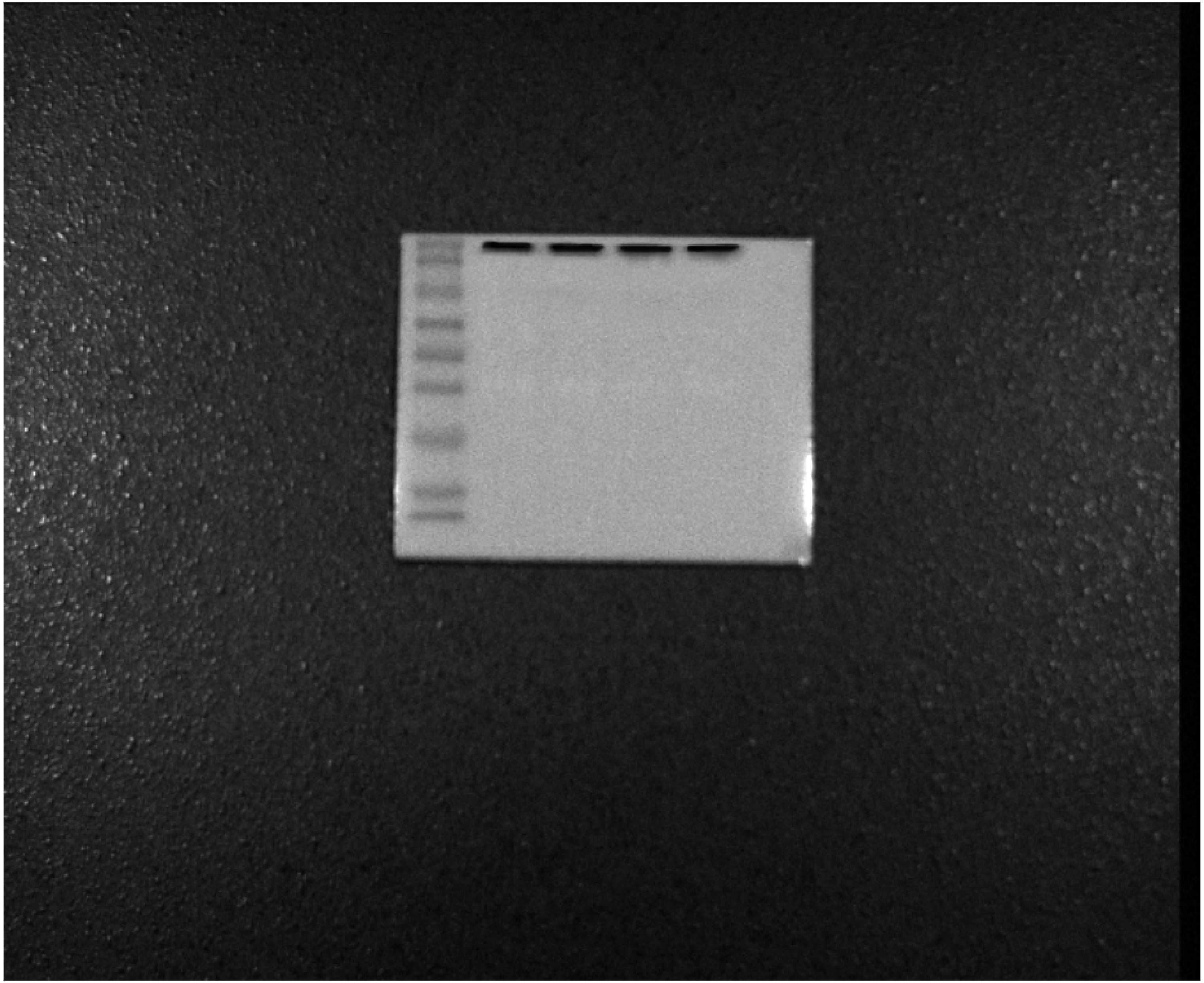

Figure 5A mTOR (2)

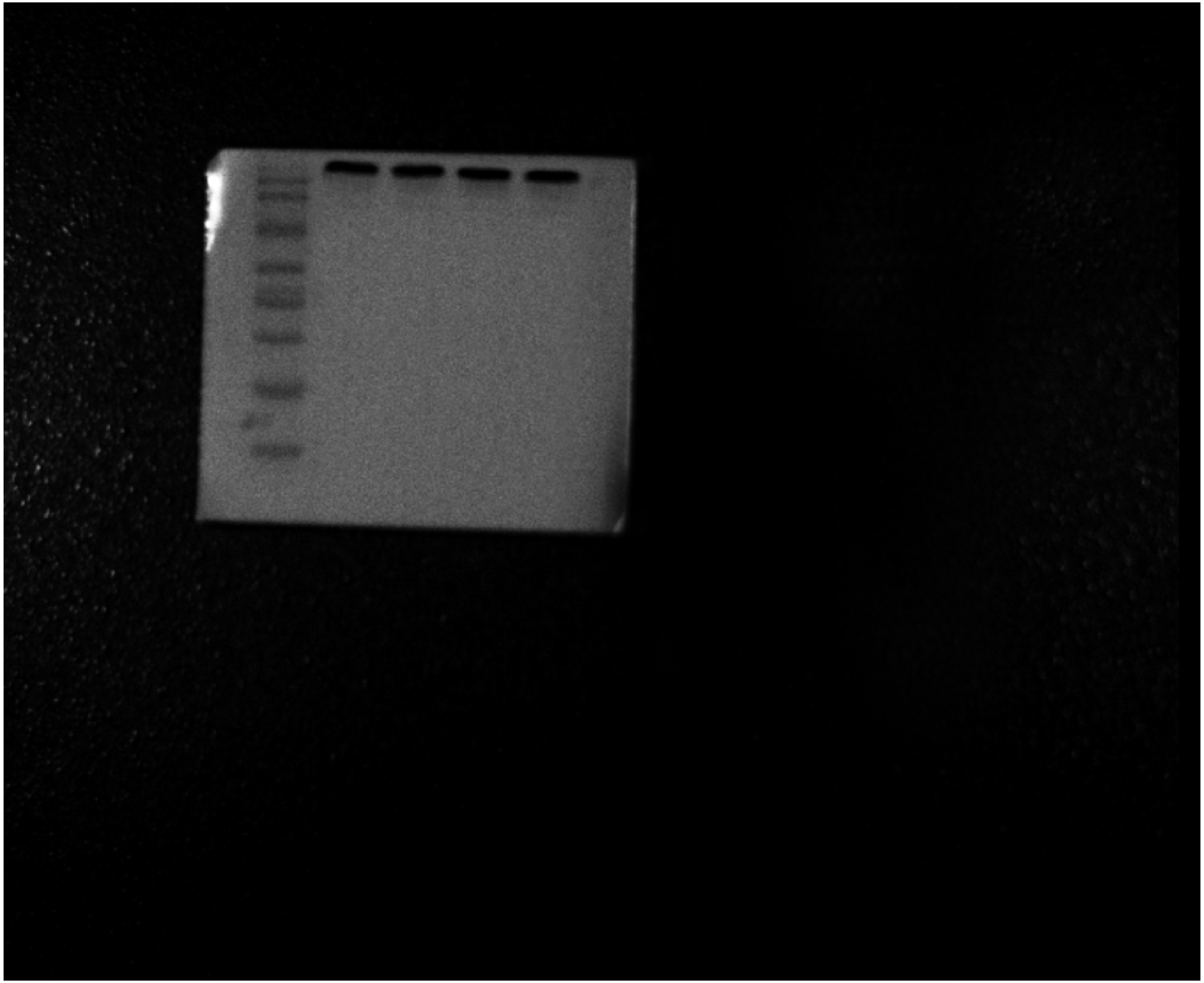

Figure 5A mTOR (3)

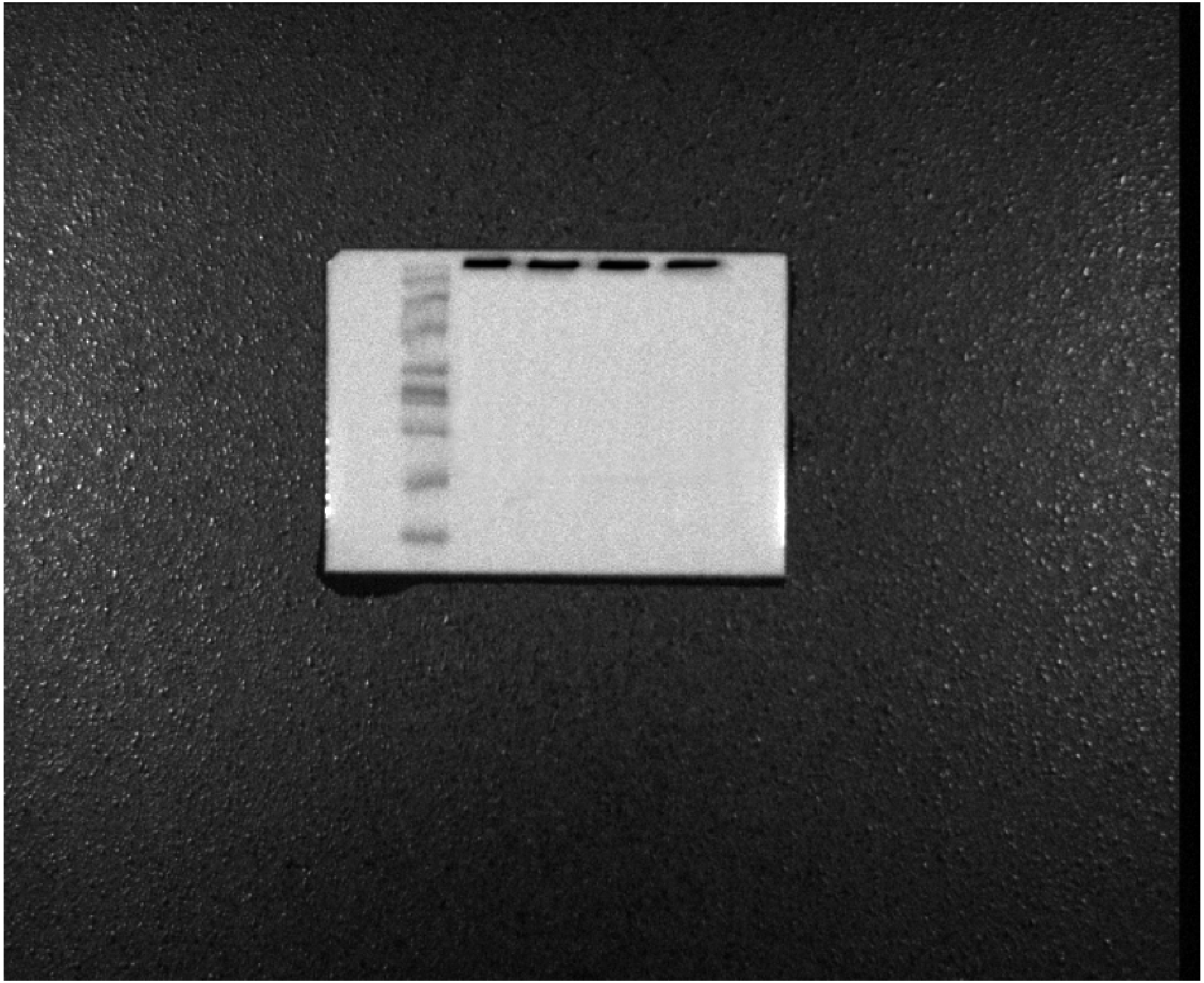

Figure 5A GAPDH (1)

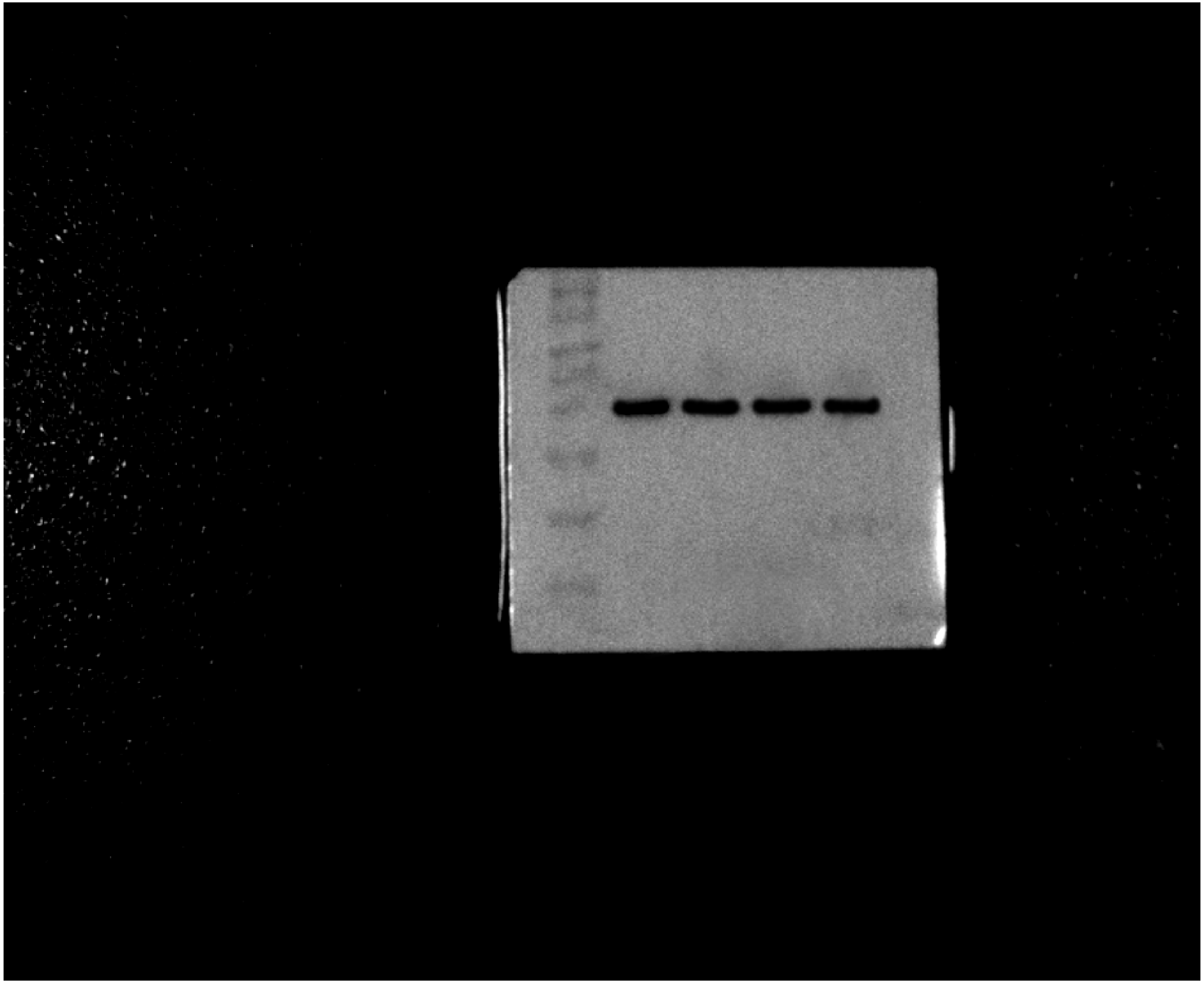

Figure 5A GAPDH (2)

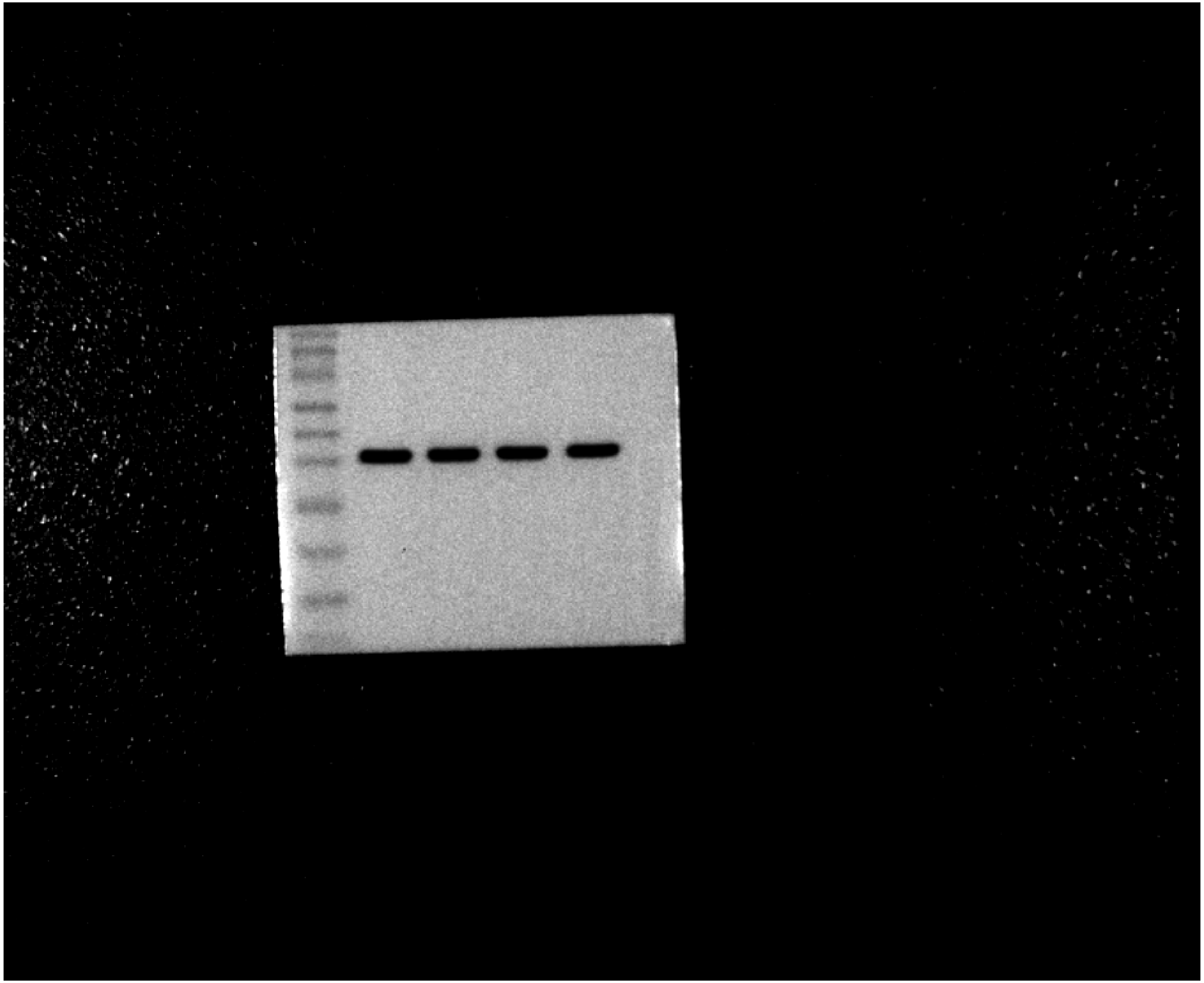

Figure 5A GAPDH (3)

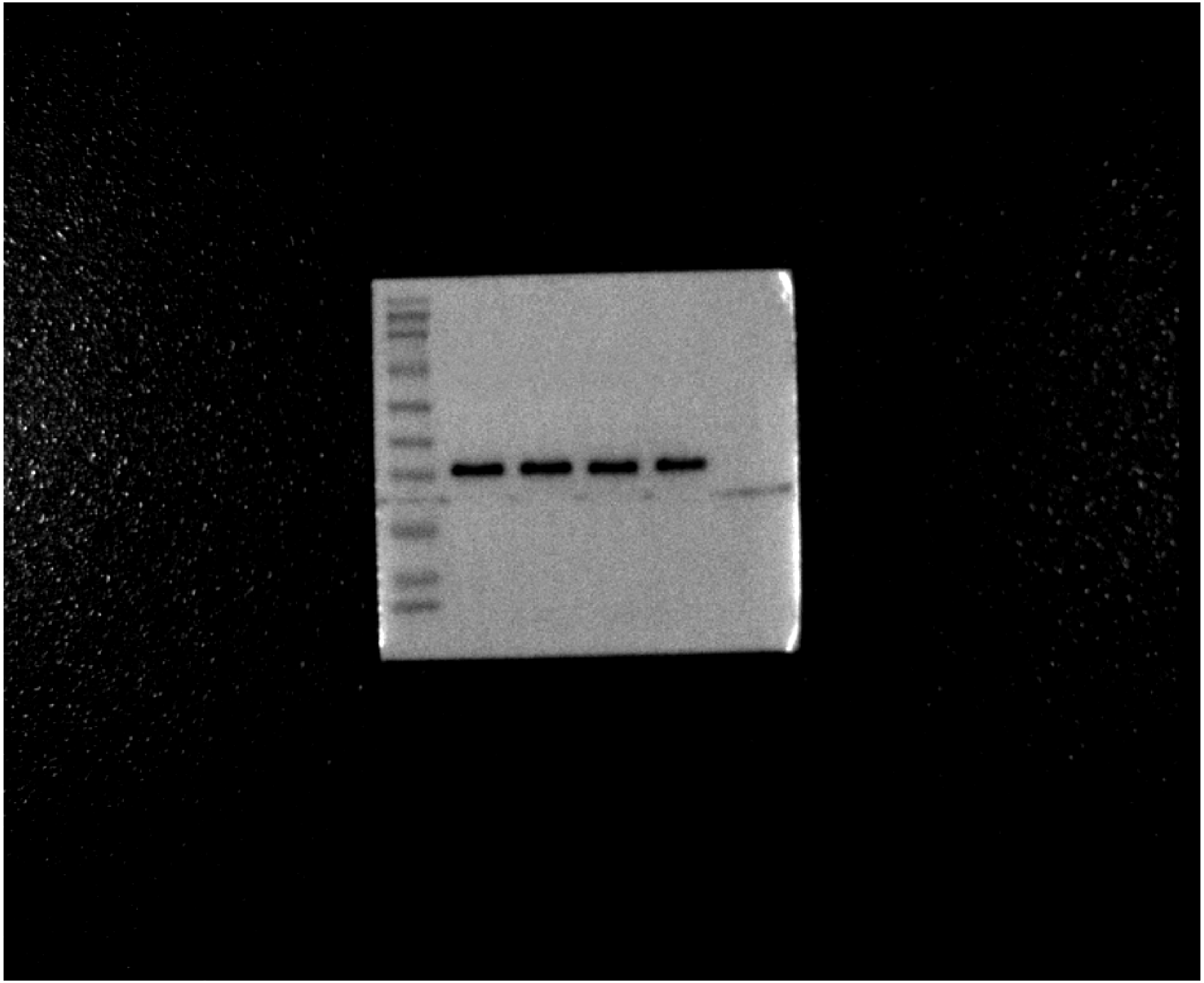

Supplement: Supplementary file 1 — Additional file 1. [file 12871_2022_1646_MOESM1_ESM.pdf]
